# Translational Block in Stroke: A Constructive and “Out-of-the-Box” Reappraisal

**DOI:** 10.3389/fnins.2021.652403

**Published:** 2021-05-14

**Authors:** Athanasios Lourbopoulos, Iordanis Mourouzis, Christodoulos Xinaris, Nefeli Zerva, Konstantinos Filippakis, Angelos Pavlopoulos, Constantinos Pantos

**Affiliations:** ^1^Department of Pharmacology, Medical School, National and Kapodistrian University of Athens, Athens, Greece; ^2^Department of Neurointensive Care Unit, Schoen Klinik Bad Aibling, Bad Aibling, Germany; ^3^Institute for Stroke and Dementia Research, Klinikum der Universität München, Ludwig Maximilian University, Munich, Germany; ^4^IRCCS – Istituto di Ricerche Farmacologiche ‘Mario Negri’, Centro Anna Maria Astori, Bergamo, Italy; ^5^University of Nicosia Medical School, Nicosia, Cyprus

**Keywords:** failure of translation, translational block, stroke, translational success, interdisciplinary, clinical, preclinical, experimental stroke models

## Abstract

Why can we still not translate preclinical research to clinical treatments for acute strokes? Despite > 1000 successful preclinical studies, drugs, and concepts for acute stroke, only two have reached clinical translation. This is the translational block. Yet, we continue to routinely model strokes using almost the same concepts we have used for over 30 years. Methodological improvements and criteria from the last decade have shed some light but have not solved the problem. In this conceptual analysis, we review the current status and reappraise it by thinking “out-of-the-box” and over the edges. As such, we query why other scientific fields have also faced the same translational failures, to find common denominators. In parallel, we query how migraine, multiple sclerosis, and hypothermia in hypoxic encephalopathy have achieved significant translation successes. Should we view ischemic stroke as a “chronic, relapsing, vascular” disease, then secondary prevention strategies are also a successful translation. Finally, based on the lessons learned, we propose how stroke should be modeled, and how preclinical and clinical scientists, editors, grant reviewers, and industry should reconsider their routine way of conducting research. Translational success for stroke treatments may eventually require a bold change with solutions that are outside of the box.

## Introduction – The Problem of Translational Failure in Stroke

Stroke remains the third leading cause of death in industrialized countries (Dirnagl et al., 1999). The literature is saturated by >1000 effective preclinical studies in acute stroke research (O’Collins et al., 2006), yet almost none are successfully transferred to the acute clinical routine. This is the well-known translational failure or block in stroke research (Endres et al., 2008).

The “basket” of stroke translational failure so far includes neuroprotective agents (O’Collins et al., 2006), stem cells (Borlongan, 2019), or even novel immunological treatments (Elkins et al., 2017), despite rigorous or/and large preclinical effect sizes. Yet we keep on modeling acute stroke and experimenting using, in most cases, the same concepts based on widely cited models. Is this the right way to continue and progress or do we simply need to critically reassess how we model ischaemic stroke?

The fact is that many pathophysiological principles of stroke were actually discovered in translation (Dirnagl and Endres, 2014). Failed clinical trials for acute stroke were probably based on rather weak preclinical evidence or inappropriate models (Drieu et al., 2020). If we consider stroke a “chronic, relapsing” disease, with multiple repeating small or large ischemic insults, then secondary prevention counts for several successes (Kernan et al., 2014). On the other hand, the differences between preclinical rodent modeling and clinical routine practice in human acute stroke are significant (Corbett et al., 2015). Nevertheless, preclinical research has several translational success stories, such as the case of experimental autoimmune encephalomyelitis (EAE) – multiple sclerosis (MS), migraine, and hypothermia in hypoxic-ischaemic encephalopathy (HIE). In addition, existing recommendation papers and consortiums [e.g., ARRIVE (Kilkenny et al., 2010), STAIR (Corbett et al., 2017), STEPS (Savitz et al., 2011)] have repeatedly proposed pathways to success. However, either few groups worldwide are implementing these guidelines or we are still missing some of the factors that are involved in failure.

Hence, we provide here a critical, interdisciplinary, and revisionary overview of stroke translational failure, taking into consideration lessons from “success stories” and “failed concepts” in neuro-research. We argue that translational failure is also the rule in ischaemia of other organs, such as myocardial infarction. Collectively, we believe that translational failure probably lies in fundamental components and “false” choices in the laboratory and clinical research. If we want to succeed, we need to improve not only the current technical hurdles in contemporary neurosciences, but also the way we put our question into perspective (Kola and Landis, 2004; Duda et al., 2014; Garner, 2014; Alteri and Guizzaro, 2018).

*“We can’t solve problems by using the same kind of thinking we used when we created them.”*

*Albert Einstein (1879–1955)*

## Translational Roadblock in Brain Ischaemia: Summarizing the Current Status, Understanding the Problem

The translational block in acute stroke has two key aspects. One is the inability of effective preclinical treatments to successfully reach clinical practice in humans. The other is the failure of clinical advances and routine therapeutic applications in Stroke Units to reach and change the way stroke is modeled in preclinical research routine. To put it differently, the blockage lies in missing or ineffective cross-talk between the preclinical side of stroke (which works with cell cultures, cells slides, rodents, and on rare occasions primates, and is usually guided by mechanism-based data for discoveries; Box 1, the typical rodent stroke “patient”), and the clinical side of stroke (which treats patients, designs and runs clinical trials and is guided by clinical and evidence-based data for decisions; Box 1, the typical human stroke patient). In pragmatic terms (Box 1), the typical stroke clinician is rarely a translational scientist and does not incorporate non-”translational” preclinical concepts into clinical routine and decision-making processes for patients. Similarly, the typical neurobiologist is rarely familiar with the clinical reality and clinical needs of stroke and rarely translates clinical advances into experimental routine (Yarborough and Dirnagl, 2017).

**BOX 1. The typical preclinical and clinical “patients” with ischaemic brain stroke.**

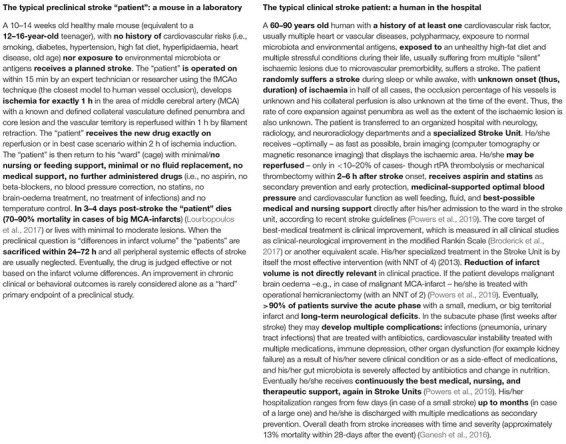

Preclinically, *in vivo* modeling of ischaemic stroke is mainly conducted in rodents (mice and rats) via three main experimental approaches (intraluminal, extraluminal, and chemical), summarized in detail in Table 1 (pros/cons, technical pitfalls, and translational significance) and extensively reviewed elsewhere (Carmichael, 2005; Howells et al., 2010; Fluri et al., 2015). Among all rodent models, the filament – or thrombus – MCAo (fMCAo or thMCAo) model and – under certain conditions – the photothrombotic model fulfill the prerequisite for “closed sterile head injury” and are probably closer to human ischaemic stroke. Additionally, the fMCAo and thMCAo are also the only ones that model recent recanalization advances in stroke. Stroke modeling in large animals (Table 2), such as non-human primates, ovine, porcine, and canine – though rarely used, in only a few selected laboratories in the world due to significant practical and ethical restrictions – possesses significant advantages as a “second species” in the translation pipeline for stroke therapies (extensively reviewed in Cai and Wang, 2016; Sorby-Adams et al., 2018; Kaiser and West, 2020). Others have also provided evidence that the rabbit small clot embolic stroke model (RSCEM) may correlate better with human brain time-windows, and probably should be used before decisions are made to authorize the clinical transfer of any novel treatment (Lapchak, 2010). *In vitro* cultures or brain slices from rodents as models of hypoxia are useful only as “add-on” methods to study isolated molecular pathways and genetic expression on cell substrates, considering all the limitations of such extra-corporal methods that lack the physiological cross-talk between the brain and the body (Honegger, 2001; Lo, 2014; Sunwoldt et al., 2017). Eventually, the selection of an appropriate model and type of study (screening, discovery, mechanistic, proof-of-concept or effectiveness of new treatment) (Gannon, 2014) may determine the result of translational research (Kola and Landis, 2004; Shanks et al., 2009; Howells et al., 2010; Dalgaard, 2015). A discovery or mechanistic preclinical study on cells or small rodents, studied for 24 h post-infarct, should not *per se* “claim” translational effectiveness in a human stroke patient (Gannon, 2014).

**TABLE 1:** Available rodent (rats, mice) models of stroke.

**Experimental model**	**Advantages**	**Disadvantages**
Intraluminal filament occlusion of the MCA (fMCAo model), through ECA or CCA (Durukan and Tatlisumak, 2007; Fisher et al., 2009; Howells et al., 2010; Canazza et al., 2014; Sommer, 2017)	- Suitable for either transient or permanent ischemia - No skull opening, no dura/brain surgical lesion - No surgical disruption of lymphatic meningeal flow - Mimics precisely human ischemic stroke (exhibits penumbra, blood-brain barrier injury, inflammatory and cell death pathways) - Reperfusion is timely controllable - Easy localization of the infarct and penumbra - Modeling of thrombectomy - Mimics malignant MCA-infarct - Rich behavioral/neurological deficits - ≈14–20 min surgery duration - Mimics malignant edema after stroke - High translationality - Most used model	- Increased risk of intracranial (subarachnoid) hemorrhage (filament-dependent) - Use of anesthesia - No modeling of thromboembolism and thrombolysis - Not feasible in animals with *rete mirabile* - Significant neck surgery (vagus nerve and trachea lesion, ischemia of ECA) - High variability (from small striatal to large malignant MCA infarcts), thus large number of animals needed - High mortality (as artifact) if no post-Stroke feeding/fluid support is used - Requires good surgical skills
Type of filaments used: - (a) Heat-treated filament with rounded tip - (b) Heat/poly-L-lysine treated - (c) Silicone coated of different length (≥2 mm)	- (a) Easy to construct, robust to use, cheap, repeated use - (b) Easy to construct, robust to use, cheap, repeated use - (c) Custom-made or industry-made, easy to use, repeated use (≈5–10 animals for custom-made, ≈40 animals for industry-made), high reproducibility of strokes with low variability, practical no vessel perforation	- (a) High risk of vessel perforation and SAH, high stroke variability, older option - (b) High risk of vessel perforation and SAH, lower stroke variability - (c) Relative expensive (only for industry-made)
Distal MCA occlusion model with craniotomy (dMCAo) (Howells et al., 2010; Canazza et al., 2014; Fluri et al., 2015; Kumar and Gupta, 2016; Sommer, 2017) via - Electrocoagulation, - Ligation or - Clotting of MCA	- Reproduces mainly permanent und -under conditions- transient ischemia - Mimics pathophysiological process of the ischemic stroke (penumbra, blood-brain barrier injury, inflammation, and cell death pathways) - Low mortality rate with high long-term survival of the animals - Low variability of infarct sizes - Localized small ischemic lesion for study - Reperfusion via thrombolysis possible only in the clotting model. - ≈ 5–8 min of surgery duration	- Skull opened, dura breached, and CSF released, thus: significant artifacts added to the ischemic lesion - Affects lymphatic circulation - Affects intracranial pressure and blood-brain barrier function severely - Contaminates the ischemic lesion with meningeal and bone (skull) cellular components (fibroblasts, osteoblasts) - No reperfusion by electrocoagulation or ligation - Heat- and mechanical trauma by electrocoagulation/ligation respectively - Use of anesthesia - Temporary and minimal neurological/behavioral deficits (maximum 1–2 weeks after stroke) - Low translationality
Proximal MCAo (occlusion of the circle of Willis or the M1 part of MCA) (Howells et al., 2010)	^–^ Approaching 100% successful induction of infarction ^–^ Highly reproducible lesion size and behavioural outcomes	- Requires significant surgical skill - Significant surgical trauma due to skull-opening - CSF leak, dura-lesion (introduction of cellular contaminants) - Affects lymphatic circulation - Recanalization possible but not usual - Rarely used, highly invasive, does not model the “closed-skull aseptic infarct” of humans - Use of anaesthesia - Low translationality
Photothrombosis model (Howells et al., 2010; Labat-gest and Tomasi, 2013; Canazza et al., 2014; Kumar and Gupta, 2016; Sommer, 2017)	- Almost no surgical intervention (only skin incision and retraction) - High reproducibility of the lesions - Accurate localization of the lesions, thus targeted neurological deficits possible - No mortality - Fast, high throughput model - Chemical platelet aggregation with local thrombi formation	- “Artificially” induced infarct, microcirculation is chemically injured. - Lesions produced in this model lack penumbra formation - Pathophysiological processes do not correspond the human clinical reality: vasogenic edema and blood-brain barrier occurs within a few minutes - Use of anesthesia for skin incision - Possible chemical interference of the light-sensitive (Rose Bengal) with the brain tissue/infarct - Thrombosis generally distributed over all vessels illuminated
Endothelin-1 model (Durukan and Tatlisumak, 2007; Howells et al., 2010; Canazza et al., 2014; Sommer, 2017)	- Ischemia induced lesions can be performed in any region of the brain - Minimal surgical invasion - Low mortality of the animals - Subcortical lesions are also possible - Recovery and plasticity mechanisms in chronic stroke can be studied	- Minimal edema - Due to the presence of endothelin-1 and endothelin-1 receptors on neurons and astrocytes it induces direct effects on brain function - Duration of ischemia is not controllable ^–^ “artificially” induced infarct, not translational
Embolic stroke model (intraluminal or distal) (Durukan and Tatlisumak, 2007; Howells et al., 2010; Canazza et al., 2014; Sommer, 2017): - (a) Thromboembolic model (prepared clots) - (b) Thrombin-enhanced clotting - (c) Non-clot embolic models (micro and macrospheres)	- Technical advantages similar to fMCAo or dMCAo models respectively - Closely mimics human thromboembolic stroke - Thrombolysis is possible (only for a and b) - Offers the opportunity of evaluation of combination therapies such as thrombolytic agents and neuroprotection (only for a and b) - High translationality	- Technical disadvantages similar to fMCAo or dMCAo models respectively - Very high variability of infarcts and behavioral outcomes due to little control over occlusion sites (for a–c) - Low rate of successful induction of stroke - Not amenable to thrombolysis (c) - Technically and timely demanding model (a) due to clot pre-formation and injection into the ICA - Rodent thrombolysis requires ≈10 times more rtPA than in humans

**TABLE 2:** Large animal species stroke (Combs et al., 1990; Traystman, 2003; Watanabe et al., 2007; Boltze et al., 2008; Rink et al., 2008; Howells et al., 2010; Lapchak, 2010; Cook and Tymianski, 2011, 2012; Wells et al., 2012; Duong, 2013; Beuing et al., 2014; Hoffmann et al., 2014; Fluri et al., 2015; Cai and Wang, 2016; Sommer, 2017; Sorby-Adams et al., 2018; Kaiser and West, 2020).

**Experimental model**	**Advantages**	**Disadvantages**
Non-human primates: - Lissencephalic brains (Squirrel Monkey, Owl monkey, Marmoset, Senegal Bush Baby) - Gyrencenphalic brain (Baboon, Rhesus, Cynomegalous macaque, African Green, Vervet Monkey) Canine, Feline, Porcine, Rabbit and Ovine models	- Key features of human behavior, pathophysiology and neuroanatomy can be studied and be better defined - Higher translationality due to higher resemblance with human brain (for species with gyrencephalic brains) - Due to higher similarity to the human brain, offer the opportunity for precise functional mapping, analytical study of deep white matter tracts, vascular architecture and gray-white matter ratio via brain CT/MRI, MR spectroscopy, brain positron emission tomography (PET), *in vivo* fluorescence and ultrasound studies - Better evaluation of endovascular methods and thrombolysis mechanisms - Clinical manifestation after stroke and drug safety are analogous to humans - Rabbit models may correlate better with the human brain time-windows	- Significant ethical questions - Require ample housing facilities, specialized care, surgical expertise skills and established neurobehavioral apparatuses - Long time of reproduction and small amount of available animals - Increased cost - Restricted knowledge of metabolic and other pathophysiology features and molecular pathways following stroke in larger species
Balloon catheters	- Minimal surgical invasion - Low mortality rate of the animals - Time precise recanalization - Very effective occlusion	- Requires expensive and special surgical materials - Confined in larger species animals - Requires fluoroscopy for catheter guidance through the vessel system of the animal

Clinically, the field of stroke trials also faces pitfalls, which are also found in other diseases (Lowenstein and Castro, 2009). Systematic errors and bias, such as recruitment of patients according to their ability to consent (Lowenstein and Castro, 2009), recruitment of “ideal” patients with less severe symptoms (Hotter et al., 2017), false-positive Phase II studies due to inappropriate time window of treatment or inappropriate outcome measures, studies that may not translationally consider significant preclinical neuropathological mechanisms, and improperly designed extensive Phase III studies with negative results (Dirnagl and Endres, 2014), are just some of these. Results based on oversimplified but easy to complete clinical scales, such as the modified Rankin Scale (mRS) (Harrison et al., 2013), may be too rough to measure subtle or localized improvements per system. Still, even in the best-designed studies (to name some examples, the NXY-059 SAINT-I and -II studies, Natalizumab ACTION-1 and -2 studies, ESCAPE-NA1 study) (Lees et al., 2006; Shuaib et al., 2007; Elkins et al., 2017; Elkind et al., 2020; Hill et al., 2020), the drug under study is either administered > 1.5 h after stroke onset, or it is exposed to interactions with the other standard medical/drug interventions (e.g., alteplase, aspirin, antihypertensives).

The wide heterogeneity of types of clinical stroke, in terms of size, location, time-windows, degrees of reperfusion or collaterals, comorbidities, and the presence or not of salvageable penumbra, may eventually be the main reason for translational failure (Howells et al., 2010). Regarding the effective time window, it is known that while rodent “patients” receive complete recanalization at exactly 45–60 min (e.g., for mice) plus treatment at the beginning of or early during ischaemia, patients have to call an ambulance at home (usually with at least a 30–45 min delay), need to go through diagnostics to exclude bleeding, and, if eligible, could potentially receive reperfusion treatment at minimum 80–90 min after ischaemia onset, thrombectomy at 3–6 hours, and finally their vessels may eventually open a few hours after stroke onset (Tsivgoulis and Alexandrov, 2014). At this time-point, the ischaemic core may already be largely established (Saver, 2006; Vilela and Rowley, 2017). As such, a clinical study of a preclinical treatment may not even be close to what rodents receive in the laboratory (Box 1), meaning that patients may fall outside of the effective time window for the treatment (Moskowitz et al., 2010). In addition to the above, stroke patients typically receive a standard and most effective “best medical treatment” in Stroke Units (Stroke Unit Trialists’ Collaboration, 2013; Powers et al., 2019), an equivalent of which (Lourbopoulos et al., 2017) is usually absent from most preclinical settings or even the recent IMPROVE guidelines for conducting rodent preclinical stroke (Percie du Sert et al., 2017). This “best medical treatment” is the obligatory control group in all clinical trials and thus a pre-existing “bias” for every tested novel drug. Eventually, admitted patients also have comorbidities and receive aspirin, beta-blockers, statins, angiotensin receptor-II (AT), or angiotensin-converting enzyme (ACE)-blockers as standard therapy, all of which have significant effects on brain and body pathophysiology *per se* (Powers et al., 2019). For several common, practical reasons (cost, difficulty in reliably and reproducibly modeling, difficulty in getting appropriate resources for these experiments), such comorbidities have not commonly been widely included in preclinical rodent modeling (Garner, 2014; Dirnagl, 2016; Modo et al., 2018). As a result, rodent studies probably test their novel treatments against translationally inappropriate control groups and neglect the effects of drug-drug interactions, polypharmacy, and comorbidities on the organism under test. These in themselves create a discrepancy between “standards” in preclinical and clinical studies, responsible for translation discordances.

Taking the problem to the level of organisms, it is fact that humans and rodents do have many similarities and hundreds of conserved molecular/genetic pathways in common as mammals (for an extensive review see Dirnagl and Endres, 2014), but there are also differences that may provoke the translational roadblock (Mestas and Hughes, 2004; Mergenthaler and Meisel, 2012; Jackson et al., 2017). For example, dissimilarities in pharmacodynamic or pharmacokinetic responses and drug bioavailability may be a result of differences in molecular pathways between species (Mestas and Hughes, 2004; Schulte-Herbruggen et al., 2006; Greek and Shanks, 2011; Bolker, 2017; Stortz et al., 2017; Zeiss and Johnson, 2017; Leenaars et al., 2019). Although the identical genetic background and capacity for genetic manipulations in mice have apparent huge advantages, these have to be carefully considered when it comes to translation because humans are genetically and environmentally highly variable (Lowenstein and Castro, 2009; Garner, 2014; Steffen et al., 2016; Vina and Sanz-Ros, 2018). Moreover, at the phenotypic level, we also need to consider the differences in the size and complexity of rodent and human brains (Herculano-Houzel, 2009). Apart from the clear difference in size [approximately 5 cm^3^ for a mouse (Badea et al., 2007) compared to 1320–1510 cm^3^ for the human brain] the mouse brain is composed of approximately 90% gray matter compared to approximately 50% of the human brain (740–820 cm^3^ gray, 360–420 cm^3^ white matter, and 230–270 cm^3^ CSF; Luders et al., 2002) and exhibits an increased glucose and oxygen metabolism (Dirnagl et al., 1999; Sorby-Adams et al., 2018; Gennaro et al., 2019). As such, a human stroke occupies different proportions of gray/white matter, with significantly different glucose/oxygen local metabolism and different vulnerability (Wang et al., 2016). At the histological level, humans and rodents – despite their stated similarities – also have anatomical, functional, morphological, and genetical differences in their astrocytes (Oberheim et al., 2009) and neurons (Kalmbach et al., 2018; Hodge et al., 2019), and to a lesser extent in oligodendrocytes/myelin (Ishii et al., 2009) and microglia (Gosselin et al., 2017). These can probably make a difference in terms of survival times and response of cells in ischaemia, different responses of the remaining network in the injured brain, and differences in plasticity and recovery mechanisms (Caleo, 2015; La Rosa and Bonfanti, 2018). Additionally, they result in a different structure and functionality of neuronal networks between the two species [e.g., the specialized corticospinal tract of quadruped rodents (Corbett et al., 2017) or the networks of higher cognitive functions in humans (Vina and Sanz-Ros, 2018)], that render translation of behavioral/neurological outcomes in stroke ever more complex. In fact, non-human primates and larger animals are closer than rodents to humans in many aspects, but are not widely available due to significant costs and ethical and practical concerns in handling and housing them (Dutta and Sengupta, 2016; Yarborough et al., 2018; Kaiser and West, 2020). Evidently, these lead to the inability to measure and translate key behavioral/neurological outcomes between humans and rodents, and on the other hand create an oversimplification of outcomes in most laboratories (e.g., using the oversimplified four-level Bederson’s neurological scale to report the neurological deficits of an infarct; Lourbopoulos et al., 2008; Balkaya et al., 2013). The latter masks relevant neuroanatomical outcome data and hinders translation (Garner, 2014; Bolker, 2017).

Finally, we believe that the age of the preclinical animals is fundamental for translation. Laboratory animals (rodents) used in preclinical studies are young (usually 8–12 weeks old), raised under strictly controlled laboratory conditions (Dirnagl et al., 1999) in a specific pathogen-free environment (Rosshart et al., 2017), and on a diet optimized for high fertility and overall health (Dirnagl, 2016). It can be argued that an 8–12 weeks old mouse corresponds to an approximately 7–11-year-old human (Jackson et al., 2017). A human trial on stroke is never performed in “7–11-year-old males with identical educational levels, identical socioeconomic statuses, identical jobs, identical – almost sterile – houses with identical (locked) thermostats, identical wives, identical diets, identical exercise regimes, in the same small town, who all incidentally had the same grandfather” (Richter et al., 2009). Age alone is a risk factor for stroke (Donnan et al., 2008) and determines the function of lesion/recovery mechanisms (Wyss-Coray, 2016), both clinically (Bindawas et al., 2017) and preclinically (Popa-Wagner et al., 2007; Li et al., 2011; Rosenzweig and Carmichael, 2013). Large clinical registries indicate that on the rare occasions that stroke does occur in children up to age 18, stroke recovery is better than in adults (deVeber et al., 2017). As such, to properly model stroke before proceeding to clinical studies, we would ideally need at least middle-aged rodents (i.e., above 14 months old, corresponding to an at least 48-year-old human) and these would have to be raised and studied in a “proper,” “humanized” environment to account for at least a rich microbiome (Rosshart et al., 2017; Hamilton and Griffith, 2019) and environment (Mering and Jolkkonen, 2015; de Boer et al., 2020).

## Known Methodological Pitfalls in Preclinical/Clinical Research in Stroke. Is this Alone the Reason for Translational Failure?

The translational roadblock in stroke is overwhelming and costly (Howells et al., 2012; London and Kimmelman, 2015). Apart from the evident aforementioned modeling, clinical/preclinical and species differences, a part of the problem lies in “how stroke research topics have been chosen and how studies have been designed, conducted, analyzed, regulated, managed, disseminated, or reported,” as recently reviewed (Berge et al., 2017). Consensus papers and consortiums [e.g., ARRIVE (Kilkenny et al., 2010), STAIR (Corbett et al., 2017), STEPS (Savitz et al., 2011), and RIGOR (Lapchak et al., 2013)] have already addressed the problem extensively. However, despite a partial improvement in stroke preclinical methodology (Bahor et al., 2017), these guidelines have only had a limited impact on the way stroke preclinical research is conducted. True compliance with guidelines such as the ARRIVE criteria remains low (Hair et al., 2019). Similarly, the applied STAIR criteria (reported from 2004 to 2011) failed to produce any benefits from subsequent clinical trials that relied on these (Lapchak et al., 2013) and appear to have had no effect on anesthetic and monitoring techniques during experimental stroke in rodents (Thomas et al., 2017).

The main current methodological pitfalls in stroke research are summarized in Table 3. Large animal stroke models have the same shortcomings as rodent ones (Kringe et al., 2020). Small studies consistently give more positive results than larger ones. Study quality is inversely related to effect size (Button et al., 2013). This, combined with rather high variance (SDs typically similar to effect size), results in low statistical power and positive predictive value (Dirnagl and Endres, 2014). In parallel, exploratory studies (usually small ones that are suitable for discovering/developing new pathophysiological theories) cannot compensate for confirmatory ones (required to be powered to investigate efficient treatments with translational significance and probability) (Kimmelman et al., 2014; Huang et al., 2020). As the field is biased toward exploratory investigation (Dirnagl, 2016), the probability of false-positive results from small and inappropriately designed studies increases (Button et al., 2013). Such studies bear low external validity, i.e., there is a small chance that their results can be applied (generalized) to other situations, groups, or events (Dirnagl, 2016; du Sert et al., 2017; Patino and Ferreira, 2018), implying low translationality. Eventually, as the typical stroke patients differ substantially from their rodent “representatives” (as discussed in detail above) (Dirnagl et al., 1999; Dirnagl, 2016; Corbett et al., 2017), a preclinical study with additionally low external validity will most probably fail to translate its result in humans (Patino and Ferreira, 2018).

**TABLE 3:** Summarized methodological pitfalls in stroke research.

**Problem**	**Definition**	**Consequences for stroke research**
Internal validity	The extent to which the observed results represent the truth in the population studied and, thus, are not due to methodological errors (Patino and Ferreira, 2018)	Low internal validity equals low chances for the results to represent the truth in the population studied. As such it: - Tends to positively biased results - Increases chances for failure to translate in humans (i.e., low external validity)
Regression to the mean	Subjects in the experiment with extreme scores will tend to move towards the average, e.g., by excluding extreme values in favor of the positive result (Holman et al., 2016).	Bias to false-positive results
Pre-testing of subjects	Pre-exposure of the subjects to the tests (Dirnagl, 2016)	Unexpected impact on the result of the test due to the interaction of the subject with the pre-test and the adaptation to the test process
Detection bias	The systematic distortion of the results of a study by non-blinded experimenters (Huang et al., 2020)	Positively biased results
Performance bias	Systematic differences between groups/experiments due to changing of animal care, housing conditions, diet, group sizes per cages, cage location in-house, instruments used, failure to complete protocols during the study (Lo, 2014; Dirnagl, 2016; Huang et al., 2020)	Data and results bias
Attrition bias	Unequal occurrence and handling of deviations from protocol and loss to follow-up between treatment groups. For example, subjects dropping out of the study (e.g., unexpected death) or undefined exclusion of “outliers” (Dirnagl, 2016; Huang et al., 2020)	Bias of results towards positive ones.
Selection bias	Biased allocation of animals at the beginning or during an experiment. Here belongs the improper randomization (Sena et al., 2010a; Lo, 2014)	- Studies do not report allocation methods, randomization, and blinding assessment of outcome. - Studies have systematic differences in baseline characteristics between treatment groups
Underpowered studies	Lack of statistically adequate subject numbers per group to reliably detect if an effect truly exists. Practically, it refers to small study groups (5–15 animals) and lack of proper sample-size calculation (Sena et al., 2010a; Dirnagl and Endres, 2014; Lo, 2014).	Low-powered studies lead to overestimated magnitude of the effect (false-positive) and lower the probability of a discovery to actually reflect a true effect
Improper statistical tests	Use of wrong, improper or not-corrected for multiple comparisons statistical tests (Makin and Orban de Xivry, 2019)	False-positive statistical results
Lack of validation, replication and confirmatory studies	A result has to be validated and data need to be replicated/confirmed in independently performed and well-designed studies (Huang et al., 2020)	Exploratory studies mainly aim to produce theories and hypotheses. If not replicated/confirmed they bare low external validity for translation.

In addition, the stroke research field is characterized by publication bias (Dirnagl, 2016) or publishing under pressure (Dirnagl and Endres, 2014; Norris et al., 2018). On the one hand, and most importantly, this means that many negative experimental stroke studies are not published (Dirnagl and Endres, 2014), so that data from as many as one in seven experiments remain unpublished (Sena et al., 2010b). On the other hand, this also means there is a preference to publish based on research directions, authors’ nationalities, or institutes’ professional ranks (Liu, 2009). Publication bias – especially the “concealing” of negative studies – has been estimated to lead to an approximately 30% overestimation of published effect sizes (Dirnagl and Endres, 2014). As a study on an “innovative idea” is easier to publish than a “verification/validation study of data,” most groups focus on such “innovative idea” studies. These produce high-impact factor publications or technologies, attract further funding and increase reputation, all the while avoiding the time- and money-consuming challenge of validating existing findings. This bias is partly attributed to a highly competitive way of doing science that sometimes runs like a “business,” where success equals more and more publications (Button et al., 2013). Stroke researchers usually seek large and acute effect sizes (”quick and large”), that make for impressive publications but are far from translational reality (Ioannidis, 2005; Sena et al., 2010b; Button et al., 2013). Eventually, these positive results lead to an overestimation of the treatment’s effectiveness in meta-analyses (Liu, 2009; Martic-Kehl et al., 2012; Dirnagl and Endres, 2014; Dirnagl, 2016; Alteri and Guizzaro, 2018), which misleads science and leads to translational failure. A classic example of this is the case of NXY-059, which had positive preclinical studies and meta-analysis but the expensive and large clinical translational program (SAINT-1 and -2 trials) was a complete failure (Bath et al., 2009).

Similar methodological pitfalls also exist in other fields of science, such as multiple sclerosis and cardiovascular research (see sections below). In the former, they seem not to affect the field, in the latter they are similarly considered a hurdle, as discussed in detail in the sections to follow. So, are methodological pitfalls alone responsible for translational failure?

## What Do We Learn From the Application of Therapeutic Hypothermia in Stroke? (Comparing a Positive and a Negative Translational Story)

Methodological problems alone probably do not suffice to create translational block, because they also exist in research fields that translate successfully. Here, we compare the example of therapeutic hypothermia (TH), studied clinically in both acute ischaemic stroke (AIS) and hypoxic brain injury after cardiopulmonary arrest. Hypothermia failed translationally in ischaemic stroke (negative story) (Kuczynski et al., 2020) but succeeded in brain hypoxia after cardiopulmonary resuscitation (positive story).

Therapeutic hypothermia has not reached bedside translation in the case of large territorial AIS (Kuczynski et al., 2020). Numerous positive preclinical studies (rats and mice) report (neuro)protection via post-ischaemic moderate hypothermia (Baron, 2018; Wu et al., 2020): it decreases brain metabolism and inhibits deleterious effects of ischaemia, including excessive neuroinflammation, cytokine release, blood-brain-barrier disruption, apoptosis, and free radical production, activated matrix metalloproteinases, ion channel change, and excitotoxicity (for extended reviews see Truettner et al., 2005; Liu et al., 2016; Wu et al., 2020). Therefore, TH preserves cell and tissue integrity during and after AIS in rodents. However, the available clinical evidence shows rather neutral brain effects and negative systemic complications in humans (Schneider et al., 2017; Wu et al., 2020). Global hypothermia induces multiple and severe systemic side effects, such as shivering, cardiac arrhythmias, vasoconstriction, pneumonia, kidney dysfunction, diffuse coagulopathy, and electrolyte imbalances (Huber et al., 2019; Kuczynski et al., 2020). Moreover, the neuroprotective effects of TH are largely time-sensitive (early hypothermia is protective, late is deleterious) (Kawamura et al., 2005; Huber et al., 2019) and depend on magnitude (mild or strong), duration of application, site of application (local on brain versus systemic application), and inherent characteristics of the organism (age and comorbidities, rodent vs. human differences) (Wu et al., 2020). Importantly, large AIS actually induces systemic hypothermia (with complications) in humans (Kvistad et al., 2012) and mice (Lourbopoulos et al., 2017). As such, the current AHA/ASA guidelines advise against systemic hypothermia in human AIS (Powers et al., 2019).

In contrast to AIS, TH is successfully used as neuroprotection after cardiac arrest and cardiopulmonary resuscitation (CPR) in adults or in neonatal hypoxic-ischaemic encephalopathy (HIE) in humans (Tagin et al., 2012; Uchino et al., 2016; Yildiz et al., 2017). The mechanisms of TH-neuroprotection are practically the same as AIS. Brain hypoxia results in abrupt energy depletion and eventually neuronal cell death, as in AIS; TH reduces brain metabolism, the toxic cascades and eventually brain damage in animal preclinical studies (Thoresen et al., 1996a,b; Ma et al., 2012; Patel et al., 2015; Shah et al., 2017; Doman et al., 2018). The prerequisites for the successful application of TH in HIE have already been defined preclinically in rodents: rapid and early onset of cooling after the hypoxic-ischaemic event (Thoresen et al., 1996b; Doman et al., 2018), prolonged duration, for several hours (3–12 h) (Thoresen et al., 1996b), reduction in rectal temperature by 4–6°C (Thoresen et al., 1996b), efficacy with both selective head cooling or whole-body hypothermia (Ma et al., 2012; Tagin et al., 2012). Here, clinical translation proved successful: TH reduces morbidity/mortality and neurological deficits in full-term infants with neonatal HIE (Jacobs et al., 2011; Tagin et al., 2012; Douglas-Escobar and Weiss, 2015; Silveira and Procianoy, 2015a,b; Purkayastha et al., 2016; Chiang et al., 2017; Giesinger et al., 2017; Rao et al., 2017; Yildiz et al., 2017; Carreras et al., 2018) as well in adults with HIE after cardiopulmonary arrest (Hypothermia after Cardiac Arrest Study Group, 2002). TH for HIE is strongly indicated – according to the latest guidelines – for adults (Panchal et al., 2020) and full-term infants (Aziz et al., 2020).

So why are the effects of TH in AIS and HIE different? In principle, both AIS and HIE receive similar clinical protocols of TH and the underlying neuroprotective mechanisms of TH are basically the same. Thus, failure or success may depend on differences between the underlying pathogenesis of hypoxia-ischaemia in AIS and HIE (Saver, 2006; Donnan et al., 2008; Douglas-Escobar and Weiss, 2015; Yildiz et al., 2017). First, the subjects in neonatal preclinical and clinical studies on HIE are very similar (same age, without comorbidities and similar – optimum – brain recovery and regeneration capacities for both species); they have high external validity, and translation succeeds. In adult HIE the age/comorbidities of rodents and humans may differ: rodent subjects in the successful preclinical studies are usually young and healthy; the effects of TH in older humans (>65 years old) are still positive but weaker (Ahn et al., 2018). Still, translation succeeds, but only under specific conditions. In AIS, age, comorbidities, and the regeneration capacity of the brain differ substantially between humans and rodents (low external validity); here, translation fails. Second, AIS and HIE probably differ in terms of the frequency and susceptibility of cortical spreading depressions (CSDs) that are triggered by and cause tissue hypoxia (Dreier, 2011; Dreier and Reiffurth, 2015). Although CSDs are established in the post-ischaemic period of AIS in animals and humans (Dreier and Reiffurth, 2015), CSD appears to be absent during the post-resuscitation period of HIE (Hansen et al., 2017), although more studies are needed. An absent CSD in HIE would imply more salvageable tissue compared to AIS. Finally, the duration of ischaemic/hypoxic injury and time of TH application determines its effect. As “time is brain” (Saver, 2006), it determines the extent of salvageable tissue and affected neuronal types (Sims, 1992; Katchanov et al., 2003) and thus their potential to be rescued. Additionally, intra-ischaemically administered TH is more effective compared to post-ischaemic cooling (Krieger and Yenari, 2004). *In vitro* models show a linear time-dependent progression of neuronal death in hypoxia (le Feber et al., 2016); thus, short ischaemia/hypoxia equals reversible damage. In transient ischaemic attacks (TIA), initial lesions are reversible due to the short duration of ischaemia. In large territorial AIS, the long duration of focal ischaemia (>1 h) “produces” irreversible necrotic injuries (infarct core), some tissue-at-risk, and initiates secondary toxic cascades (e.g., release of DAMPs, cortical spreading depressions and aseptic inflammation) that aggravate damage (Moskowitz et al., 2010; Iadecola et al., 2020); here, TH fails. In the case of HIE, brief hypoxia (several minutes) with restoration of circulation causes diffuse “tissue-at-risk” with no necrotic core; it resembles TIA. Its reversible injury depends strictly on the duration of the insult (Douglas-Escobar and Weiss, 2015; Uchino et al., 2016; Elmer and Callaway, 2017; Sekhon et al., 2017). When HIE is relatively short, TH can improve cerebral metabolism and act protectively by decreasing free radical production, inflammation, excitotoxicity, and intracranial pressure (Darwazeh and Yan, 2013). As such, TH is clinically highly effective in both adults (Hypothermia after Cardiac Arrest Study Group, 2002; Geocadin et al., 2006) and newborns (Jacobs et al., 2013) after a relatively fast “return-of-spontaneous-circulation” (ROSC). On the other hand, prolonged cardiac arrest (≥40 min) aggravates neuronal injury to an irreversible injury with a worse prognosis (Welbourn and Efstathiou, 2018). Indeed, an indirect comparison of two study cohorts treated with TH after cardiac arrest shows there were good neurological outcomes in 18% of patients with 40 min CPR versus 55% of patients with 22 min CPR, confirming the time-sensitive protective effect of TH (Hypothermia after Cardiac Arrest Study Group, 2002; Ahn et al., 2018).

As such, differences in the ages of subjects, the presence of secondary cascades such as CSD and duration of hypoxia/ischaemia in AIS/HIE determine the success or failure for translation of hypothermia. Methodological problems in preclinical studies seem to play only a minor role.

## Same Problem in a Different Organ: Translational Roadblock in Heart Ischaemia

The translational block is not a “privilege” of neurology alone but also exists in other scientific fields with analogies that are worth considering (Box 2).

**BOX 2. The typical preclinical and clinical “patients” with a heart attack.**

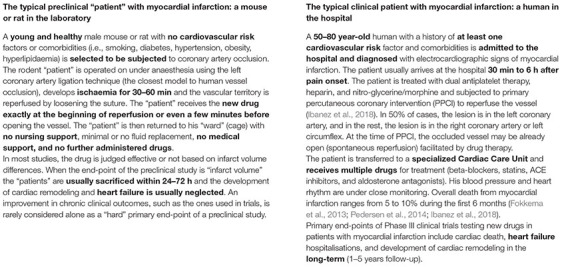

In the case of heart ischaemia, the fact is that despite timely reperfusion, morbidity and mortality following ST-elevation myocardial infarction (STEMI) remain substantial (Fokkema et al., 2013; Pedersen et al., 2014; Ibanez et al., 2018). Despite applied reperfusion techniques and optimal pharmacotherapy, almost half of all patients will develop structural and functional heart failure (van der Bijl et al., 2020), while nearly 30% of patients will additionally develop clinical signs and symptoms of heart failure within 1 year (Velagaleti et al., 2008; Ambrosy et al., 2014) (Box 2: the typical human patient with “heart attack”). Here, cardioprotection in patients with acute myocardial infarction parallels neuroprotection in ischaemic stroke. It aims to reduce myocardial injury, the attenuation of heart failure development, and to improve survival and quality of life. However, the translation of thousands of preclinical studies on mechanical and pharmacological cardioprotective interventions in these patients has been particularly disappointing over the last 30 years (Heusch, 2013, 2017). Different cardioprotective strategies, such as coronary postconditioning, hypothermia, GP IIb/IIIa inhibitors, drugs targeting nitric oxide pathways, cyclosporine, adenosine, erythropoietin, glucose modulators, and others, have shown successful preclinical results but all failed in clinical trials to improve patient outcomes (Ludman et al., 2011; Heusch, 2019). Typical examples of translational failure are large and expensive Phase III clinical trials with thousands of STEMI patients, where the primary intervention (e.g., remote ischaemic conditioning) cannot reduce either infarct size or cardiac mortality and hospitalization for heart failure (Hausenloy et al., 2019).

As such, the translation of cardioprotection from bench to bedside remains a significant challenge. Regarding cardiology, the differences between the human patient with acute myocardial infarction (AMI) and the corresponding animal models are at the core of this problem (Box 2: the typical rodent “patient” with “heart attack”). The experimental studies are mostly performed in young and otherwise healthy animals without comorbidities and cardiovascular risk factors that cause endothelial dysfunction. On the other hand, humans with AMI usually have several comorbidities (diabetes, hypothyroidism, hypertension) and risk factors (smoking, hypercholesterolemia) that interfere with cardioprotective interventions (Ferdinandy et al., 2014). Smoking is related to reduced efficacy of remote ischaemic conditioning to improve infarct size (Sloth et al., 2015). In diabetes and hypothyroidism, hearts are found to be resistant to the protective effect of ischaemic conditioning (Pantos et al., 2004; Kleinbongard et al., 2019). Furthermore, cardioprotective strategies may become redundant due to several routine medications that are used in patients with AMI and may reduce myocardial injury *per se* (Heusch and Gersh, 2017). These medications include β-blockers, statins, nitrates, antiplatelets, and opioids (Pugsley, 2002; Ferdinandy et al., 2014). A retrospective analysis showed that antiplatelets (P2Y_12_ antagonists) may abrogate the effect of ischaemic postconditioning on infarct size reduction (Roubille et al., 2012). On the other hand, medications such as insulin or anti-diabetic drugs and ACE inhibitors may also induce cardioprotection. The effect of sex is often neglected in experimental studies. Most studies are performed in male animals, while female patients with AMI may have a worse prognosis (Cenko et al., 2018).

Apart from the above problems, a crucial issue is the definition of the proper inclusion-exclusion criteria in clinical trials to include patients with AMI that may benefit from cardioprotective intervention. The existence of salvageable myocardium (in correlation to penumbra in the brain) is important and has to do mostly with cases reperfused 1–6 h after the onset of symptoms (Gersh et al., 2005; Heusch and Gersh, 2017). A small myocardium infarct usually leads to a low risk for heart failure. If reperfusion is very early, then myocardial injury will be negligible and cardioprotection is redundant. If reperfusion is late, little salvageable myocardium will remain (Gersh et al., 2005; Heusch and Gersh, 2017). “Time is heart” in the same way that “time is brain.” Factors such as the presence of pre-infarction angina (it functions like ischaemic preconditioning and results in minimal infarcts; Kleinbongard et al., 2019) or the presence of myocardial injury that occurs during the first few minutes of reperfusion (Rossello and Yellon, 2016) may also change the final outcome and have to be addressed in study designs (Bøtker et al., 2010). On top of that, the selection of the proper dose and timing of administration of cardioprotective interventions may be crucial for successful translation. Unfortunately, in most cases, adequate Phase II pharmacokinetic trials that aim to define the effective time and dose are missing. On the other hand, experimental studies rarely determine effective drug concentrations or routes in blood or serum. For example, adenosine reduces myocardial infarction after reperfusion only when a high dose and prolonged intracoronary infusion of the drug is used (Yetgin et al., 2015).

Infarct size reduction has been the focus of nearly all experimental studies on cardioprotection. However, clinical reality based on recent trials indicates that this focus may have been too narrow (Heusch, 2019). There is no doubt that infarct size is a determinant of patients‘ prognosis (Stone et al., 2016) but other important factors may independently determine the prognosis of patients with reperfused STEMI. In contrast to infarct size, the long-term effects of cardioprotective interventions on myocardial function, repair, remodeling, and mortality have not been thoroughly investigated in preclinical studies (Heusch, 2017). The recent examples of cyclosporine and remote ischaemic conditioning are indicative. In several preclinical and small clinical studies, both interventions proved efficient in reducing infarct size but the large Phase III clinical trials were completely neutral regarding mortality, heart failure hospitalisations, and cardiac remodeling at 1 year (Cung et al., 2015; Hausenloy et al., 2019). Furthermore, besides infarct size, other important factors of myocardial injury, such as microvascular obstruction (MVO) have been neglected in experimental studies. Clinical data show that any evidence of MVO in patients with reperfused AMI is associated with poorer prognostic outcomes, more adverse remodeling, and lower ventricular function on follow-up (Borlotti et al., 2019; Mangion et al., 2019) and with more cardiovascular events, notably higher mortality on follow-up (van Kranenburg et al., 2014; Reinstadler et al., 2018; Galea et al., 2019). Indeed, only MVO but not infarct size predicted cardiac mortality after 2 years of follow-up (van Kranenburg et al., 2014). Eventually, reassessment of the primary end-points that we set in experimental studies seems necessary to produce data with more translational value in cardiology.

## Looking at the Bright Side of Translation: The Successful Examples of Multiple Sclerosis and Migraine

On the other hand, translation does work in some fields of neuroscience. In our opinion, the best examples of this are Experimental Autoimmune Encephalomyelitis (EAE) and Multiple Sclerosis (MS) (t Hart et al., 2018) along with the development of CGRP-targeted drugs for migraine (Wattiez et al., 2019). Despite existing failures (Rolfes et al., 2020), EAE led to the development of multiple drugs for MS that control its inflammatory component, such as INF-β, fingolimod, fumarate, mitoxantrone, cladribine, teriflunomide, glatiramer acetate, siponimod, and antibodies such as ocrelizumab, natalizumab, rituximab, and alemtuzumab (Grzegorski and Losy, 2019; Yamout et al., 2020). Migraine preclinical research resulted in targeted CGRP-antagonists and antibodies for migraine (Edvinsson et al., 2018; Charles and Pozo-Rosich, 2019). Hence, what are the keys to these successes?

EAE and MS share many common features (Box 3: typical human and rodent “patients”). EAE is a collection of models, each of which reproduces different clinical, neuropathological, and immunological features of MS (but none alone reproduce complete human MS disease, for an extensive review see Gold et al., 2006; Lassmann and Bradl, 2017; Hochstrasser et al., 2018; Titus et al., 2020). Here the correct choice of model is decisive for each scientific question posed. The immune system in rodents and humans is both similar (structural) and different (molecular) (Mestas and Hughes, 2004). The two diseases share several immunological and pathological pathways and mechanisms (immune-mediated demyelination and axonal injury) that serve well for pathomechanistic understanding and drug discoveries (for extensive and detailed reviews please see Steinman and Zamvil, 2005; Gold et al., 2006; Lassmann and Bradl, 2017; Bjelobaba et al., 2018; Glatigny and Bettelli, 2018). Such corresponding common features can also be found in the field of stroke (pathomechanisms, structure, pathways, clinic). Furthermore, studies on EAE face the same severe methodological and bias problems as stroke studies. Publication bias, insufficient sample sizes, and statistics, lack of blinding and randomisation procedures, lack of multicenter studies, lack of validation studies, etc. are widespread in the EAE field (Vesterinen et al., 2010) but these pitfalls do not block translation. However, EAE and MS have some striking similarities that do not exist in the field of ischaemic stroke. Both involve subjects of a similar age without comorbidities (young mice and patients) (Box 3), both have a decisive peripheral inflammatory component that is easy to reach via bloodstream medications, both have an extended “incubation” period for interventions before disease onset in the CNS that equals to a wide therapeutic window and the aim for both EAE and MS is to develop prophylactic treatment to prevent a relapse (or immune activation) rather than the correction of an already established lesion. Practically, such successfully “translated” MS drugs target and reach mainly the peripheral inflammatory component of the disease (Meuth et al., 2010). In that sense, the prevention of stroke is also successful through secondary prevention medications (statins, antiplatelets, antihypertensives, anticoagulation) (Hankey, 2017). When all the above parameters are considered collectively and critically, the translational block seems to arise when we try to restore/treat cellular degeneration. In other words, it seems easier to succeed translationally when treating a disease target outside of the blood-brain-barrier, in a preventive manner, with an extended therapeutic time window and in young subjects without comorbidities.

**BOX 3. The typical preclinical and clinical “patient” with multiple sclerosis.**

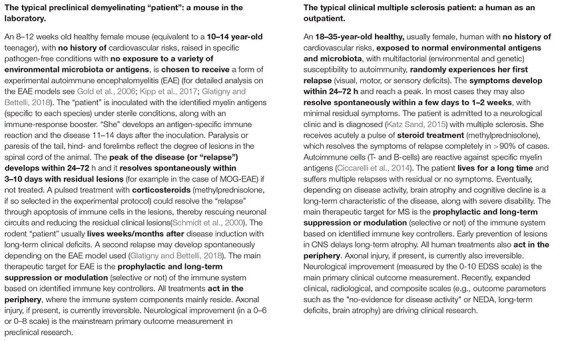

The second example is migraine, a disease that enjoys a rapidly advancing understanding of its pathophysiology (for extensive reviews please see Russell et al., 2014; Charles, 2018; Hay et al., 2018; Wattiez et al., 2019). Such knowledge comes in large from preclinical research on small animal models of migraine-related pain, as reviewed in detail (Harriott et al., 2019). Initially, research on serotonin mechanisms in migraine led to the successful and large class of triptans (Goadsby, 2005), as well as the brand new class of selective 5-HT1F agonists (Lasmiditan) (Clemow et al., 2020). In parallel, the calcitonin gene-related peptide (CGRP) pathway (Hay et al., 2018) has been studied extensively, its contribution is well characterized during pain, as CGRP is – uniquely – released during migraine (Edvinsson, 2017), its release into the cranial venous outflow during migraine attacks is proven and intravenous CGRP can induce migraine-like symptoms in migraine patients (Edvinsson, 2017) (direct cause-effect connection). As such, the related CGRP-targeted therapies tested for its treatment to date have consistently produced positive results (Edvinsson, 2017; Schuster and Rapoport, 2017): CGRP-receptor antagonists (gepants) and antibodies (erenumab, galcanezumab, fremanezumab, and eptinezumab) currently being developed are well tolerated and constitute a useful therapeutic tool (Steiner et al., 2013; Edvinsson, 2015; Schuster and Rapoport, 2016; May, 2017; Edvinsson et al., 2018; Charles and Pozo-Rosich, 2019). In addition, studies on new migraine-associated genes, visualization of early activated brain regions just before a migraine attack (e.g., hypothalamic and brainstem activation in collaboration with cortical spreading depolarisation), and the role for neuropeptides on pain (e.g., Pituitary adenylate cyclase-activating polypeptide, PACAP) (Rubio-Beltran et al., 2018) pave the path for new prophylactic options for migraine (Pascual, 2015; Charles, 2018; Harriott et al., 2019). It seems that the field of migraine research is characterized by clearly defined molecular pathways with a crystal-clear cause-effect connection, the age of preclinical and clinical subjects is similar, comorbidities are absent, the targeted pathway is not “behind” the blood-brain barrier (Miller et al., 2016), the primary clinical target is functional (symptom control of viable tissue) rather than anatomical (no evident tissue lesion) and treatment is prophylactic. Most of these features are common denominators in the aforementioned successful MS-EAE paradigm as well.

## Seeking “Out-Of-The-Box” Solutions for the Current Common Stroke Practice and Translation Pipeline

Based on the above argumentation, the translationability of our acute therapeutic strategies could be assumed to correlate linearly with the capacity of our models to mimic human acute ischemic physiology: in other words, the more reliably we mimic the human body at the bench the more efficient our drugs will be at the bedside.

In our opinion, the first step should be preclinical testing of drugs on human tissue, after initial screening in rodents and before the transfer of potent drugs to clinical studies. Rodent cells cannot always mimic one-to-one the effects of drugs on human cells, reflecting cellular differences between species. One striking example of this is the neuroprotectant NXY-059, which was a success in rat models but a complete failure in clinical settings, and was recently shown to offer no protection when tested on stem cell-derived human neurons (Antonic et al., 2018). To this end, tissue engineering and stem cell technologies could provide the next-generation human tissue substrate for drug testing. Stem cell-derived 3D engineered tissues or organoids can efficiently mimic key features of the original organ and have been used successfully to model certain facets of human organogenesis and disease, personalized drug testing, and discovery studies. (reviewed in Li and Izpisua Belmonte, 2019; Xinaris, 2019; Steimberg et al., 2020). Human organoids enable the study of human developmental processes and pathogenetic pathways in multilayer and multicellular complexes, especially when the pathways involve interactions between different cell types of the organ. For instance, brain human organoids have been used to examine cell division orientation in human radial glial cells (Lancaster et al., 2013) and human cortical progenitor expansion (Otani et al., 2016) – both processes significantly different in humans compared to other species and especially rodents (Lancaster et al., 2013; Otani et al., 2016). Nevertheless, organoids still have crucial insufficiencies compared to the original human organs, such as a lack of vasculature and immune cells [for a detailed review of organoids’ limitations Ref (Xinaris, 2019)]. This means that they are limited in terms of how much they can grow without cell death and how they are influenced by circulating molecules, mechanical stress, and neural inputs. Lab-grown organoids also exhibit significant anatomic inadequacies due to the absence of normal directional cues (both biochemical and mechanical) that drive the correct organization of cells within tissues and organs (Bhaduri et al., 2020). Yet, despite these aforementioned precautions, the preclinical testing of candidate drugs on human organoids could most probably avoid failures of expensive clinical trials due to species’ cellular differences. Here, studies of “reverse translation,” i.e., testing failed drugs from humans back to rodents to compare responses under the insights provided by their relevant trials, would reveal patterns of translational success/failure and would improve our future strategy in drug development in stroke.

Maybe we should simply find what rodent model best mimics translation, or we could also “humanize” our rodent preclinical models, at least before bringing the candidate drugs to the clinic phase. Rats may eventually not be the optimal rodent species for drug translation in stroke; mice may be more “translational.” Comparative data from preclinical and clinical studies for the Natalizumab, NXY-059, Magnesium, and especially mesenchymal stem cells treatment in stroke imply that mice either reflect the negative human results or the effect size better than rats (Shuaib et al., 2007; Bath et al., 2009; Vu et al., 2014; Llovera et al., 2015; Saver et al., 2015; Elkind et al., 2020). Recent studies indicate that preclinical inbred and SPF rodents fail to recapitulate a normal “dirty” human environment due to their poor and altered microbiome, mycobiome, and virome. The restoration of the organism’s wild environment results in mice and rats (”wildlings”) that can phenocopy human immune responses due to their “wild” immune system trained by the normal environment (Beura et al., 2016; Rosshart et al., 2017, 2019; Hamilton and Griffith, 2019; de Boer et al., 2020). Such “wildings” affect the immune landscape of multiple organs, bring them closer to human status and are also more resistant to diseases compared to inbred laboratory ones (Rosshart et al., 2017). As the immune post-stroke local and systemic reactions largely dictate and shape neuroplasticity and regeneration after stroke (Iadecola et al., 2020), such a “wild” immune system could provide a “human” background for preclinical stroke rodent studies. Furthermore, mice can be “humanized” after being engrafted with human myeloid, lymphoid cell lineages, and tissues (Rongvaux et al., 2014; Bryce et al., 2016; Ito et al., 2018) or via the introduction of human genes that do not exist in mice (“humanized” knock in-mouse models) (Casas et al., 2019). Studies from other research fields indicate that such mouse strains could be biologically closer to humans with higher translational validity, proven so far in cancer immunotherapy, regenerative medicine, allergy and graft-versus-host disease (Ito et al., 2018). Given that the immune system is key to degeneration and regeneration processes after ischaemic stroke, the aforementioned “humanized” strains could bridge the gap between preclinical models and stroke patients.

However, preclinical and clinical fields can be also bridged through computational approaches, such as Machine Learning (ML) and Neural Networks (NN) or study design web-platforms such as the Experimental Design Assistant (EDA) and study-reporting databases. Here, computational science can dig through the vast web-information or “omics” data provided nowadays and reveal similarities or resolve discrepancies between species that result in successful or failed translation (Brubaker et al., 2019). The challenge is to simply predict human biology from non-human species. In the Found In Translation project^1^, a ML model applied on gene expression datasets from mouse models of 28 different human diseases achieved an improvement in predictions for human phenotype by 20–50%, compared to simple and direct cross-species extrapolation (Normand et al., 2018). According to the authors, this approach highlights signals that may otherwise be missed and – most importantly – reduces false leads, and thus could enhance translational output for stroke at no additional costs (Normand et al., 2018). Alternative approaches used microarray datasets (from trauma, burn, sepsis) to train a neural network in order to identify true human biological associations after interpreting mouse experiments (Brubaker et al., 2019) or used data from human genome-wide disease studies combined with *in silico* network models of tissue- and cell-specific function in model organisms to spot candidate molecules that are functionally conserved between rodent-human species (Yao et al., 2018). Interestingly, all achieved truer human biological associations, while mouse experiments alone (i.e., “traditional translation”) failed to capture a large portion of human *in vivo* biology (Brubaker et al., 2019). Additionally, recently developed preclinical study design assistants, such as the Experimental Design Assistant can help researchers improve the design of animal experiments (du Sert et al., 2017). We believe that such platforms can significantly facilitate preclinical stroke study quality. They should be used widely, and the produced flow-diagrams of each study should be included as figures in manuscripts. Moreover, the preclinical stroke studies should be registered for transparency on websites such as https://www.preclinicaltrials.eu/ or https://www.animalstudyregistry.org, as is routinely done for clinical trials (FDA-based website^2^). Such registries enhance proper study design, validity, and the reproducibility of experiments/data while simultaneously securing intellectual property.

Finally, we strongly suggest that the drug discovery pipeline in stroke should include at least one validation study of the results in aged animals, optimally in a multicenter design and under co-administration of alteplase. The importance of aging in the biology of the organism is extensively discussed above. STAIR criteria highlight this (Fisher et al., 2009). Data from research fields other than stroke strongly imply that differences in age may be the key to things being lost translation in stroke. To name a few examples, old microglia respond weakly compared to young ones (Daria et al., 2017), aging alters cerebral arteries’ neurovascular coupling in mice (Balbi et al., 2015), aging alters cortex energy and the redox metabolism of the brain (Bayliak et al., 2021), aging changes the response to bacterial endophthalmitis, aging alters the immunological response to stroke (Ritzel et al., 2018), heart systolic function is decreased in older rats (Raya et al., 1997). As aged animals and multicenter preclinical trials can be costly (Llovera et al., 2015), we suggest running one multicenter trial in aged rodents as the “decision-maker” before proceeding to the (more expensive) Phase II clinical stroke studies. As such, in case of negative results, a failed transfer to the clinical setup can be avoided. A typical example of this concept is the negative anti-CD49d preclinical multicenter study in fMCAo in mice (Llovera et al., 2015) that was recently “replicated” with similar negative results in two large Phase II clinical trials (ACTION-1 and -2) (Elkins et al., 2017; Elkind et al., 2020). Eventually, preclinical stroke research should test novel compounds for interactions with alteplase before going to the clinic. In addition to its beneficial thrombolytic action, Alteplase has neurotoxic side effects (Kaur et al., 2004; Dong et al., 2016) and may mask positive results in stroke clinical trials when thrombolysis is used as the standard reperfusion option in treatment-placebo arms (Hankey, 2020). This suggestion is based on the recent ESCAPE-NA1 trial that studied the neuroprotectant nerinetide in human ischaemia-reperfusion that occurs with rapid endovascular thrombectomy (Hill et al., 2020). The ESCAPE-NA1 trial was optimally translationally designed based on a preclinical development program following STAIR criteria and studies on rodents and non-human primates (Hankey, 2020; Hill et al., 2020). Subgroup analysis indicated that patients under only mechanical thrombectomy but not alteplase-thromboysis benefit from nerinetide, supporting a “toxic” masking effect of alteplase in this group.

## Conclusion: Opinion Statement and Suggested Path for Translation

**FIGURE 1:**
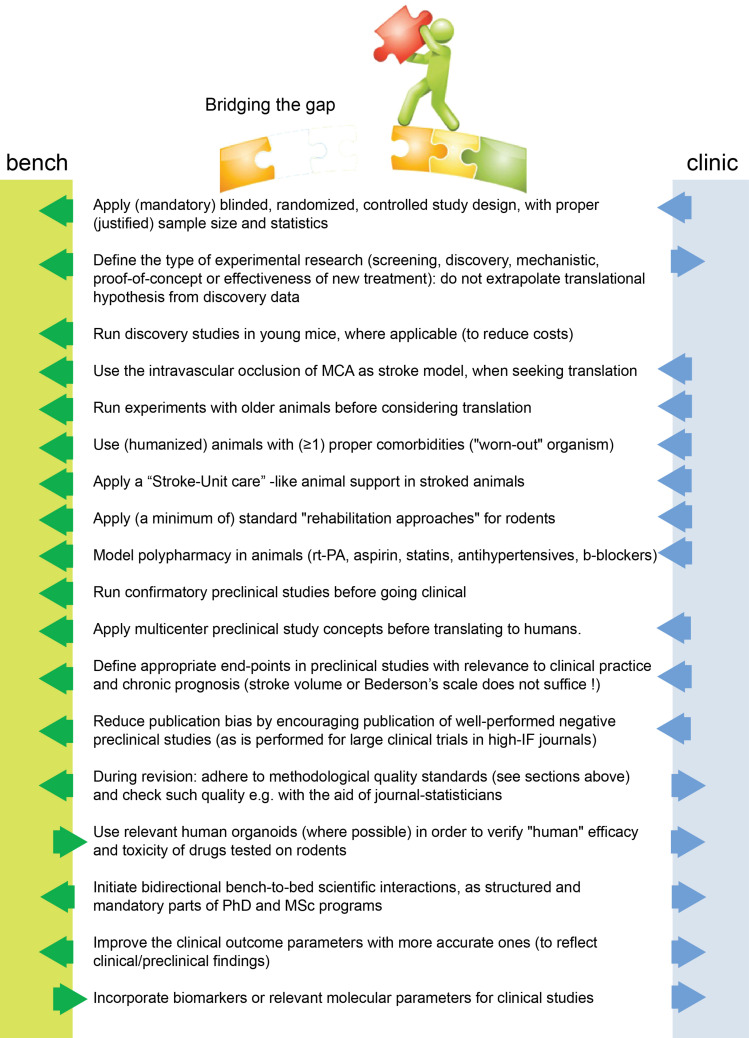
Suggested changes for stroke translation. Arrows (blue and green) point in the direction that each measure should be applied for improved translation.

In conclusion, translation seems to work mainly at the mechanistic/discovery level. For example, the mechanisms of excitotoxicity, periinfarct depolarisations, inflammation, glymphatic system, and programmed cell death have been discovered in rodents and have also been verified in humans (Dirnagl et al., 1999; Moskowitz et al., 2010; Gennaro et al., 2019; Mestre et al., 2020). On the other side, if we consider stroke as a “chronic” vascular disease with “relapses” of small or large ischemic insults, then its secondary prevention is also a translational success (Kernan et al., 2014), as applies for the case of relapse-prevention in MS as well. Under this assumption, translational failure is a problem only of the acute/subacute phase of stroke, or, named otherwise, a problem of neuroprotective and/or neuroregenerative efforts. Eventually, to overcome this blockage for acute/subacute treatments we believe we need to bridge concepts and practices from both pre- and clinical sides of stroke research (Figure 1) and introduce concepts that arise from research fields other than brain stroke as well.

Collectively, we believe that methodological and statistical pitfalls alone do not block acute translation. It remains disappointing that despite the ARRIVE (Kilkenny et al., 2010), STAIR (Corbett et al., 2017), and STEPS(Savitz et al., 2011) guidelines for *in vivo* experimentation, very few preclinical manuscripts have achieved full compliance with these. Similar suggestions have come from other fields, such as the field of preclinical cardioprotective therapies (Jones et al., 2015; Lindsey et al., 2018). We should not neglect the fact that the only translated and clinically applied treatment to human stroke is the recanalization of the occluded vessel *per se* (thrombectomy or thrombolysis) (Powers et al., 2019), when we model thrombectomy preclinically in the fMCAo model (Carmichael, 2005). And this is repeated over decades, despite methodological and statistical pitfalls.

**FIGURE 2:**
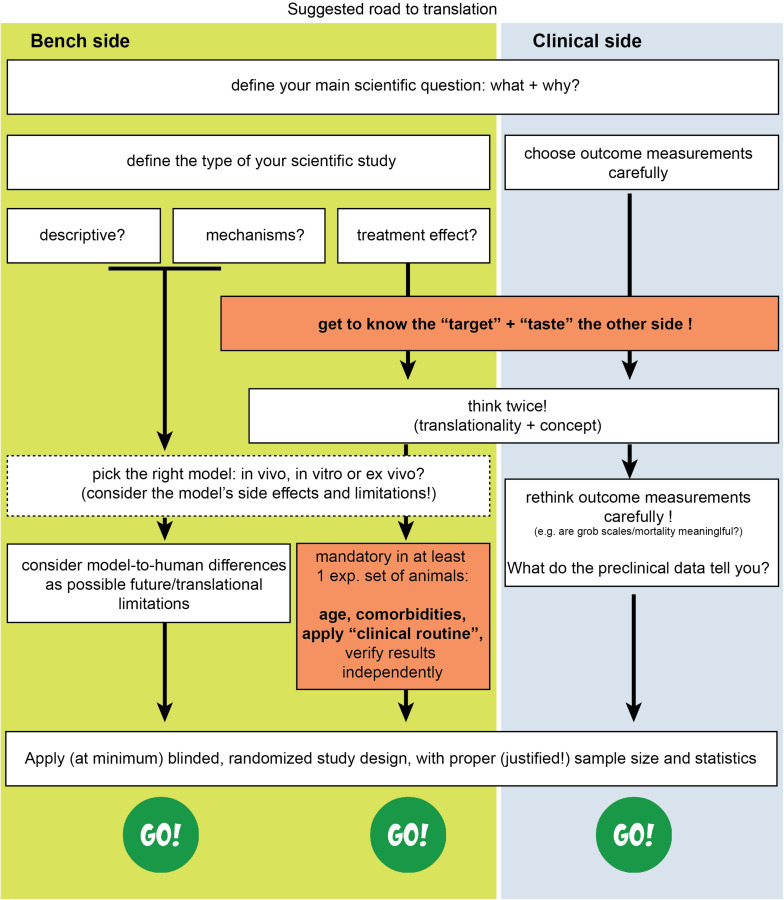
Suggested road to translational success in stroke. The missing effective cross-talk between the basic neuroscience side (bench side) and the clinical neurology side (clinical side) is a key reason for failure.

Therefore, in addition to existing suggested guidelines (Kilkenny et al., 2010; Savitz et al., 2011; Mergenthaler and Meisel, 2012; Corbett et al., 2017), we believe that we need to think about acute stroke research differently, as summarized in Figures 1, 2. We need preclinical research conditions that mimic the acute clinical ones: a “stroke unit” and “best medical treatment” support for rodents with aspirin, statin, alteplase, treatment of infections, fluids, nutritional support (even if this means negative preclinical results) (Lourbopoulos et al., 2017), and standard translational “rehabilitation approaches” for rodents, such as enriched environments (Garner, 2014; Winek et al., 2016; Bolker, 2017). We need to bring our rodent “patients” closer to the “reality” of human patients: middle-aged to elderly patients, with vascular comorbidities (worn-out vessels, “multi-hits” concept of a chronic pathophysiology) and western-diet, an intravascular (embolic or thrombotic) model of stroke, at least some degree of reperfusion, with an intact skull. We need to incorporate computational aids to design and analyze our data, run multicenter preclinical trials before going clinical, and run “reverse translational studies” (i.e., testing concepts from humans back to animals for translational validity). These are needed along with the high methodological quality of preclinical research that should be guaranteed and accessed via a preclinical trials registry and structured review process by the journals. For this, journals and reviewers hold the key to implementation. Success will come only when we bridge current standards between bench and bedside, conduct quality research and think boldly “out-of-the-box” of the current routine.

## Data Availability Statement

The raw data supporting the conclusions of this article will be made available by the authors, without undue reservation.

## Author Contributions

AL conceived, wrote, and critically reviewed the manuscript. IM and CX wrote and critically reviewed the manuscript. NZ, KF, and AP wrote sections of the manuscript. CP critically reviewed and supervised the manuscript. All the authors contributed to the article and approved the submitted version.

## Conflict of Interest

The authors declare that the research was conducted in the absence of any commercial or financial relationships that could be construed as a potential conflict of interest.

## Footnotes

1 http://www.mouse2man.org

2 clinicaltrials.gov

## References

[B1] Ahn S. Lee B. K. Youn C. S. Kim Y. J. Sohn C. H. Seo D. W. (2018). Predictors of good neurologic outcome after resuscitation beyond 30 min in out-of-hospital cardiac arrest patients undergoing therapeutic hypothermia. *Intern. Emerg. Med.* 13 413–419. 28389857 10.1007/s11739-017-1662-4

[B2] Alteri E. Guizzaro L. (2018). Be open about drug failures to speed up research. *Nature* 563 317–319. 10.1038/d41586-018-07352-7 30425369

[B3] Ambrosy A. P. Fonarow G. C. Butler J. Chioncel O. Greene S. J. Vaduganathan M. (2014). The global health and economic burden of hospitalizations for heart failure: lessons learned from hospitalized heart failure registries. *J. Am. Coll. Cardiol.* 63 1123–1133. 24491689 10.1016/j.jacc.2013.11.053

[B4] Antonic A. Dottori M. Macleod M. R. Donnan G. A. Howells D. W. (2018). NXY-059, a failed stroke neuroprotectant, offers no protection to stem cell-derived human neurons. *J. Stroke Cerebrovasc. Dis.* 27 2158–2165. 10.1016/j.jstrokecerebrovasdis.2018.03.015 29673616

[B5] Aziz K. Lee H. C. Escobedo M. B. Hoover A. V. Kamath-Rayne B. D. Kapadia V. S. (2020). Part 5: neonatal resuscitation: 2020 American heart association guidelines for cardiopulmonary resuscitation and emergency cardiovascular care. *Circulation* 142 S524–S550. 33081528 10.1161/CIR.0000000000000902

[B6] Badea A. Ali-Sharief A. A. Johnson G. A. (2007). Morphometric analysis of the C57BL/6J mouse brain. *Neuroimage* 37 683–693. 10.1016/j.neuroimage.2007.05.046 17627846 PMC2176152

[B7] Bahor Z. Liao J. Macleod M. R. Bannach-Brown A. Mccann S. K. Wever K. E. (2017). Risk of bias reporting in the recent animal focal cerebral ischaemia literature. *Clin. Sci.* 131 2525–2532. 10.1042/cs20160722 29026002 PMC5869854

[B8] Balbi M. Ghosh M. Longden T. A. Jativa Vega M. Gesierich B. Hellal F. (2015). Dysfunction of mouse cerebral arteries during early aging. *J. Cereb. Blood Flow Metab.* 35 1445–1453. 10.1038/jcbfm.2015.107 26058694 PMC4640303

[B9] Balkaya M. Krober J. M. Rex A. Endres M. (2013). Assessing post-stroke behavior in mouse models of focal ischemia. *J. Cereb. Blood Flow Metab.* 33 330–338. 10.1038/jcbfm.2012.185 23232947 PMC3587814

[B10] Baron J. C. (2018). Protecting the ischaemic penumbra as an adjunct to thrombectomy for acute stroke. *Nat. Rev. Neurol.* 14 325–337. 10.1038/s41582-018-0002-2 29674752

[B11] Bath P. M. Gray L. J. Bath A. J. Buchan A. Miyata T. Green A. R. (2009). Effects of NXY-059 in experimental stroke: an individual animal meta-analysis. *Br. J. Pharmacol.* 157 1157–1171. 10.1111/j.1476-5381.2009.00196.x 19422398 PMC2743834

[B12] Bayliak M. M. Sorochynska O. M. Kuzniak O. V. Gospodaryov D. V. Demianchuk O. I. Vasylyk Y. V. (2021). Middle age as a turning point in mouse cerebral cortex energy and redox metabolism: Modulation by every-other-day fasting. *Exp. Gerontol.* 145:111182. 10.1016/j.exger.2020.111182 33290862

[B13] Berge E. Al-Shahi Salman R. Van Der Worp H. B. Stapf C. Sandercock P. Sprigg N. (2017). Increasing value and reducing waste in stroke research. *Lancet Neurol.* 16 399–408. 28414653 10.1016/S1474-4422(17)30078-9

[B14] Beuing O. Boese A. Kyriakou Y. Deuerling-Zengh Y. Jöllenbeck B. Scherlach C. (2014). A novel technique for the measurement of CBF and CBV with robot-arm-mounted flat panel CT in a large-animal model. *Am. J. Neuroradiol.* 35 1740–1745. 10.3174/ajnr.a3973 24831590 PMC7966271

[B15] Beura L. K. Hamilton S. E. Bi K. Schenkel J. M. Odumade O. A. Casey K. A. (2016). Normalizing the environment recapitulates adult human immune traits in laboratory mice. *Nature* 532 512–516. 10.1038/nature17655 27096360 PMC4871315

[B16] Bhaduri A. Andrews M. G. Mancia Leon W. Jung D. Shin D. Allen D. (2020). Cell stress in cortical organoids impairs molecular subtype specification. *Nature* 578 142–148. 10.1038/s41586-020-1962-0 31996853 PMC7433012

[B17] Bindawas S. M. Vennu V. Mawajdeh H. Alhaidary H. (2017). Functional outcomes by age after inpatient stroke rehabilitation in Saudi Arabia. *Clin. Interv. Aging* 12 1791–1797. 10.2147/cia.s145402 29123384 PMC5661488

[B18] Bjelobaba I. Begovic-Kupresanin V. Pekovic S. Lavrnja I. (2018). Animal models of multiple sclerosis: focus on experimental autoimmune encephalomyelitis. *J. Neurosci. Res.* 96 1021–1042. 10.1002/jnr.24224 29446144

[B19] Bolker J. A. (2017). Animal models in translational research: rosetta stone or stumbling block? *Bioessays* 39 10.1002/bies.201700089 29052843

[B20] Boltze J. Förschler A. Nitzsche B. Waldmin D. Hoffmann A. Boltze C. M. (2008). Permanent middle cerebral artery occlusion in sheep: a novel large animal model of focal cerebral ischemia. *J. Cereb. Blood Flow Metab.* 28 1951–1964. 10.1038/jcbfm.2008.89 18698332

[B21] Borlongan C. V. (2019). Concise review: stem cell therapy for stroke patients: are we there yet? *Stem Cells Transl. Med.* 8 983–988. 10.1002/sctm.19-0076 31099181 PMC6708064

[B22] Borlotti A. Jerosch-Herold M. Liu D. Viliani D. Bracco A. Alkhalil M. (2019). Acute microvascular impairment post-reperfused STEMI is reversible and has additional clinical predictive value: a CMR OxAMI study. *JACC Cardiovasc. Imaging* 12 1783–1793. 10.1016/j.jcmg.2018.10.028 30660541 PMC6718360

[B23] Bøtker H. E. Kharbanda R. Schmidt M. R. Bøttcher M. Kaltoft A. K. Terkelsen C. J. (2010). Remote ischaemic conditioning before hospital admission, as a complement to angioplasty, and effect on myocardial salvage in patients with acute myocardial infarction: a randomised trial. *Lancet* 375 727–734. 10.1016/s0140-6736(09)62001-8 20189026

[B24] Broderick J. P. Adeoye O. Elm J. (2017). Evolution of the modified rankin scale and its use in future stroke trials. *Stroke* 48 2007–2012. 10.1161/strokeaha.117.017866 28626052 PMC5552200

[B25] Brubaker D. K. Proctor E. A. Haigis K. M. Lauffenburger D. A. (2019). Computational translation of genomic responses from experimental model systems to humans. *PLoS Comput. Biol.* 15:e1006286. 10.1371/journal.pcbi.1006286 30629591 PMC6343937

[B26] Bryce P. J. Falahati R. Kenney L. L. Leung J. Bebbington C. Tomasevic N. (2016). Humanized mouse model of mast cell-mediated passive cutaneous anaphylaxis and passive systemic anaphylaxis. *J. Allergy Clin. Immunol.* 138 769–779. 10.1016/j.jaci.2016.01.049 27139822 PMC5014665

[B27] Button K. S. Ioannidis J. P. A. Mokrysz C. Nosek B. A. Flint J. Robinson E. S. J. (2013). Power failure: why small sample size undermines the reliability of neuroscience. *Nat. Rev. Neurosci.* 14 365–376. 10.1038/nrn3475 23571845

[B28] Cai B. Wang N. (2016). Large animal stroke models vs. rodent stroke models, pros and cons, and combination? *Acta Neurochir. Suppl.* 121 77–81. 10.1007/978-3-319-18497-5_13 26463926

[B29] Caleo M. (2015). Rehabilitation and plasticity following stroke: insights from rodent models. *Neuroscience* 311 180–194. 10.1016/j.neuroscience.2015.10.029 26493858

[B30] Canazza A. Minati L. Boffano C. Parati E. Binks S. (2014). Experimental models of brain ischemia: a review of techniques, magnetic resonance imaging, and investigational cell-based therapies. *Front. Neurol.* 5:19. 10.3389/fneur.2014.00019 24600434 PMC3928567

[B31] Carmichael S. T. (2005). Rodent models of focal stroke: size, mechanism, and purpose. *NeuroRx* 2 396–409. 10.1602/neurorx.2.3.396 16389304 PMC1144484

[B32] Carreras N. Alsina M. Alarcon A. Arca-Diaz G. Agut T. Garcia-Alix A. (2018). Efficacy of passive hypothermia and adverse events during transport of asphyxiated newborns according to the severity of hypoxic-ischemic encephalopathy. *J. Pediatr.* 94 251–257. 10.1016/j.jpedp.2017.08.025 28822711

[B33] Casas A. I. Kleikers P. W. Geuss E. Langhauser F. Adler T. Busch D. H. (2019). Calcium-dependent blood-brain barrier breakdown by NOX5 limits postreperfusion benefit in stroke. *J. Clin. Invest.* 129 1772–1778. 10.1172/jci124283 30882367 PMC6436900

[B34] Cenko E. Yoon J. Kedev S. Stankovic G. Vasiljevic Z. Krljanac G. (2018). Sex differences in outcomes after STEMI: effect modification by treatment strategy and age. *JAMA Intern. Med.* 178 632–639. 10.1001/jamainternmed.2018.0514 29630703 PMC6145795

[B35] Charles A. (2018). The pathophysiology of migraine: implications for clinical management. *Lancet Neurol.* 17 174–182. 10.1016/s1474-4422(17)30435-0 29229375

[B36] Charles A. Pozo-Rosich P. (2019). Targeting calcitonin gene-related peptide: a new era in migraine therapy. *Lancet* 394 1765–1774. 10.1016/s0140-6736(19)32504-8 31668411

[B37] Chiang M.-C. Jong Y.-J. Lin C.-H. (2017). Therapeutic hypothermia for neonates with hypoxic ischemic encephalopathy. *Pediatr. Neonatol.* 58 475–483. 28416250 10.1016/j.pedneo.2016.11.001

[B38] Ciccarelli O. Barkhof F. Bodini B. De Stefano N. Golay X. Nicolay K. (2014). Pathogenesis of multiple sclerosis: insights from molecular and metabolic imaging. *Lancet Neurol.* 13 807–822. 10.1016/s1474-4422(14)70101-2 25008549

[B39] Clemow D. B. Johnson K. W. Hochstetler H. M. Ossipov M. H. Hake A. M. Blumenfeld A. M. (2020). Lasmiditan mechanism of action - review of a selective 5-HT1F agonist. *J. Headache Pain* 21:71. 10.1186/s10194-020-01132-3 PMC7288483 32522164

[B40] Combs D. J. Dempsey R. J. Kumar S. Donaldson D. (1990). Focal cerebral infarction in cats in the presence of hyperglycemia and increased insulin. *Metab. Brain Dis.* 5 169–178. 10.1007/bf00997070 2087216

[B41] Cook D. J. Tymianski M. (2011). Translating promising preclinical neuroprotective therapies to human stroke trials. *Expert Rev. Cardiovasc. Ther.* 9 433–449. 10.1586/erc.11.34 21517728

[B42] Cook D. J. Tymianski M. (2012). Nonhuman primate models of stroke for translational neuroprotection research. *Neurotherapeutics* 9 371–379. 10.1007/s13311-012-0115-z 22437447 PMC3337022

[B43] Corbett D. Carmichael S. T. Murphy T. H. Jones T. A. Schwab M. E. Jolkkonen J. (2017). Enhancing the alignment of the preclinical and clinical stroke recovery research pipeline: consensus-based core recommendations from the stroke recovery and rehabilitation roundtable translational working group. *Int. J. Stroke* 12 462–471. 10.1177/1747493017711814 28697710

[B44] Corbett D. Jeffers M. Nguemeni C. Gomez-Smith M. Livingston-Thomas J. (2015). Lost in translation: rethinking approaches to stroke recovery. *Prog. Brain Res.* 218 413–434. 25890148 10.1016/bs.pbr.2014.12.002

[B45] Cung T. T. Morel O. Cayla G. Rioufol G. Garcia-Dorado D. Angoulvant D. (2015). Cyclosporine before PCI in patients with acute myocardial infarction. *N. Engl. J. Med.* 373 1021–1103. 26321103 10.1056/NEJMoa1505489

[B46] Dalgaard L. (2015). Comparison of minipig, dog, monkey and human drug metabolism and disposition. *J. Pharmacol. Toxicol. Methods* 74 80–92. 10.1016/j.vascn.2014.12.005 25545337

[B47] Daria A. Colombo A. Llovera G. Hampel H. Willem M. Liesz A. (2017). Young microglia restore amyloid plaque clearance of aged microglia. *EMBO J.* 36 583–603. 10.15252/embj.201694591 28007893 PMC5331757

[B48] Darwazeh R. Yan Y. (2013). Mild hypothermia as a treatment for central nervous system injuries: positive or negative effects. *Neural Regen. Res.* 8 2677–2686. 25206579 10.3969/j.issn.1673-5374.2013.28.010 PMC4146029

[B49] de Boer A. Storm A. Gomez-Soler M. Smolders S. Rue L. Poppe L. (2020). Environmental enrichment during the chronic phase after experimental stroke promotes functional recovery without synergistic effects of EphA4 targeted therapy. *Hum. Mol. Genet.* 29 605–617. 10.1093/hmg/ddz288 31814004 PMC7068116

[B50] deVeber G. A. Kirton A. Booth F. A. Yager J. Y. Wirrell E. C. Wood E. (2017). Epidemiology and outcomes of arterial ischemic stroke in children: the Canadian pediatric ischemic stroke registry. *Pediatr. Neurol.* 69 58–70. 10.1016/j.pediatrneurol.2017.01.016 28254555

[B51] Dirnagl U. (2016). Thomas willis lecture: is translational stroke research broken, and if so, how can we fix it? *Stroke* 47 2148–2153. 10.1161/strokeaha.116.013244 27354221

[B52] Dirnagl U. Endres M. (2014). Found in translation: preclinical stroke research predicts human pathophysiology, clinical phenotypes, and therapeutic outcomes. *Stroke* 45 1510–1518. 10.1161/strokeaha.113.004075 24652307

[B53] Dirnagl U. Iadecola C. Moskowitz M. A. (1999). Pathobiology of ischaemic stroke: an integrated view. *Trends Neurosci.* 22 391–397. 10.1016/s0166-2236(99)01401-0 10441299

[B54] Doman S. E. Girish A. Nemeth C. L. Drummond G. T. Carr P. Garcia M. S. (2018). Early detection of hypothermic neuroprotection using T2-weighted magnetic resonance imaging in a mouse model of hypoxic ischemic encephalopathy. *Front. Neurol.* 9:304. 10.3389/fneur.2018.00304 29867720 PMC5951924

[B55] Dong M. X. Hu Q. C. Shen P. Pan J. X. Wei Y. D. Liu Y. Y. (2016). Recombinant tissue plasminogen activator induces neurological side effects independent on thrombolysis in mechanical animal models of focal cerebral infarction: a systematic review and meta-analysis. *PLoS One* 11:e0158848. 10.1371/journal.pone.0158848 27387385 PMC4936748

[B56] Donnan G. A. Fisher M. Macleod M. Davis S. M. (2008). Stroke. *Lancet* 371 1612–1623. 18468545 10.1016/S0140-6736(08)60694-7

[B57] Douglas-Escobar M. Weiss M. D. (2015). Hypoxic-ischemic encephalopathy: a review for the clinician. *JAMA Pediatr.* 169 397–403. 10.1001/jamapediatrics.2014.3269 25685948

[B58] Dreier J. P. (2011). The role of spreading depression, spreading depolarization and spreading ischemia in neurological disease. *Nat. Med.* 17 439–447. 10.1038/nm.2333 21475241

[B59] Dreier J. P. Reiffurth C. (2015). The stroke-migraine depolarization continuum. *Neuron* 86 902–922. 10.1016/j.neuron.2015.04.004 25996134

[B60] Drieu A. Buendia I. Levard D. Helie P. Brodin C. Vivien D. (2020). Immune responses and anti-inflammatory strategies in a clinically relevant model of thromboembolic ischemic stroke with reperfusion. *Transl. Stroke Res.* 11 481–495. 10.1007/s12975-019-00733-8 31522409

[B61] du Sert N. P. Bamsey I. Bate S. T. Berdoy M. Clark R. A. Cuthill I. C. (2017). The experimental design assistant. *Nat. Methods* 14 1024–1025. 28960183 10.1038/nmeth.4462 PMC7610684

[B62] Duda G. N. Grainger D. W. Frisk M. L. Bruckner-Tuderman L. Carr A. Dirnagl U. (2014). Changing the mindset in life sciences toward translation: a consensus. *Sci. Transl. Med.* 6:264cm212. 10.1126/scitranslmed.aaa0599 25429054

[B63] Duong T. Q. J. N. R. (2013). Magnetic resonance imaging of perfusion–diffusion mismatch in rodent and non-human primate stroke models. *Neurol. Res.* 35 465–469. 10.1179/1743132813y.0000000211 23594679 PMC3715998

[B64] Durukan A. Tatlisumak T. (2007). Acute ischemic stroke: overview of major experimental rodent models, pathophysiology, and therapy of focal cerebral ischemia. *Pharmacol. Biochem. Behav.* 87 179–197. 10.1016/j.pbb.2007.04.015 17521716

[B65] Dutta S. Sengupta P. (2016). Men and mice: relating their ages. *Life Sci.* 152 244–248. 10.1016/j.lfs.2015.10.025 26596563

[B66] Edvinsson L. (2015). The journey to establish CGRP as a migraine target: a retrospective view. *Headache* 55 1249–1255. 10.1111/head.12656 26368117

[B67] Edvinsson L. (2017). The trigeminovascular pathway: role of CGRP and CGRP receptors in migraine. *Headache* 57(Suppl. 2), 47–55. 10.1111/head.13081 28485848

[B68] Edvinsson L. Haanes K. A. Warfvinge K. Krause D. N. (2018). CGRP as the target of new migraine therapies - successful translation from bench to clinic. *Nat. Rev. Neurol.* 14 338–350. 10.1038/s41582-018-0003-1 29691490

[B69] Elkind M. S. V. Veltkamp R. Montaner J. Johnston S. C. Singhal A. B. Becker K. (2020). Natalizumab in acute ischemic stroke (ACTION II): a randomized, placebo-controlled trial. *Neurology* 95 e1091–e1104. 32591475 10.1212/WNL.0000000000010038 PMC7668547

[B70] Elkins J. Veltkamp R. Montaner J. Johnston S. C. Singhal A. B. Becker K. (2017). Safety and efficacy of natalizumab in patients with acute ischaemic stroke (ACTION): a randomised, placebo-controlled, double-blind phase 2 trial. *Lancet Neurol.* 16 217–226. 10.1016/s1474-4422(16)30357-x 28229893

[B71] Elmer J. Callaway C. W. (2017). The brain after cardiac arrest. *Semin. Neurol.* 37 19–24. 28147414 10.1055/s-0036-1597833 PMC6402583

[B72] Endres M. Engelhardt B. Koistinaho J. Lindvall O. Meairs S. Mohr J. P. (2008). Improving outcome after stroke: overcoming the translational roadblock. *Cerebrovasc. Dis.* 25 268–278. 10.1159/000118039 18292653

[B73] Ferdinandy P. Hausenloy D. J. Heusch G. Baxter G. F. Schulz R. (2014). Interaction of risk factors, comorbidities and comedications with ischemia/reperfusion injury and cardioprotection by preconditioning, postconditioning, and remote conditioning. *Pharmacol. Rev.* 66 1142–1174. 10.1124/pr.113.008300 25261534

[B74] Fisher M. Feuerstein G. Howells D. W. Hurn P. D. Kent T. A. Savitz S. I. (2009). Update of the stroke therapy academic industry roundtable preclinical recommendations. *Stroke* 40 2244–2250. 10.1161/strokeaha.108.541128 19246690 PMC2888275

[B75] Fluri F. Schuhmann M. K. Kleinschnitz C. (2015). Animal models of ischemic stroke and their application in clinical research. *Drug. Des. Dev. Ther.* 9 3445–3454. 10.2147/dddt.s56071 26170628 PMC4494187

[B76] Fokkema M. James S. Albertsson P. Akerblom A. Calais F. Eriksson P. (2013). Population trends in percutaneous coronary intervention: 20-year results from the SCAAR (Swedish Coronary Angiography and Angioplasty Registry). *J. Am. Coll. Cardiol.* 61 1222–1230. 23500325 10.1016/j.jacc.2013.01.007

[B77] Galea N. Dacquino G. Ammendola R. Coco S. Agati L. De Luca L. (2019). Microvascular obstruction extent predicts major adverse cardiovascular events in patients with acute myocardial infarction and preserved ejection fraction. *Eur. Radiol.* 29 2369–2377. 10.1007/s00330-018-5895-z 30552479

[B78] Ganesh A. Lindsay P. Fang J. Kapral M. K. Cote R. Joiner I. (2016). Integrated systems of stroke care and reduction in 30-day mortality: a retrospective analysis. *Neurology* 86 898–904. 10.1212/wnl.0000000000002443 26850979 PMC4782112

[B79] Gannon F. (2014). The steps from translatable to translational research. *EMBO Rep.* 15 1107–1108. 10.15252/embr.201439587 25296643 PMC4253482

[B80] Garner J. P. (2014). The significance of meaning: why do over 90% of behavioral neuroscience results fail to translate to humans, and what can we do to fix it? *ILAR J.* 55 438–456. 10.1093/ilar/ilu047 25541546 PMC4342719

[B81] Gennaro M. Mattiello A. Pizzorusso T. (2019). Rodent models of developmental ischemic stroke for translational research: strengths and weaknesses. *Neural Plast.* 2019:5089321. 10.1155/2019/5089321 PMC6476045 31093271

[B82] Geocadin R. G. Koenig M. A. Stevens R. D. Peberdy M. A. (2006). Intensive care for brain injury after cardiac arrest: therapeutic hypothermia and related neuroprotective strategies. *Crit. Care Clin.* 22 619–636; abstract viii. 17239747 10.1016/j.ccc.2006.11.008

[B83] Gersh B. J. Stone G. W. White H. D. Holmes D. R. (2005). Pharmacological facilitation of primary percutaneous coronary intervention for acute myocardial infarction: is the slope of the curve the shape of the future? *J. Am. Med. Assoc.* 293 979–986. 10.1001/jama.293.8.979 15728169

[B84] Giesinger R. E. Bailey L. J. Deshpande P. Mcnamara P. J. (2017). Hypoxic-ischemic encephalopathy and therapeutic hypothermia: the hemodynamic perspective. *J. Pediatr.* 180 22.e2–30.e2. 27742125 10.1016/j.jpeds.2016.09.009

[B85] Glatigny S. Bettelli E. (2018). Experimental autoimmune encephalomyelitis (EAE) as animal models of multiple sclerosis (MS). *Cold Spring Harb. Perspect. Med.* 8:a028977. 10.1101/cshperspect.a028977 29311122 PMC6211376

[B86] Goadsby P. J. (2005). Can we develop neurally acting drugs for the treatment of migraine? *Nat. Rev. Drug Discov.* 4 741–750. 10.1038/nrd1822 16121129

[B87] Gold R. Linington C. Lassmann H. (2006). Understanding pathogenesis and therapy of multiple sclerosis via animal models: 70 years of merits and culprits in experimental autoimmune encephalomyelitis research. *Brain* 129 1953–1971. 10.1093/brain/awl075 16632554

[B88] Gosselin D. Skola D. Coufal N. G. Holtman I. R. Schlachetzki J. C. M. Sajti E. (2017). An environment-dependent transcriptional network specifies human microglia identity. *Science* 356:eaal3222. 10.1126/science.aal3222 28546318 PMC5858585

[B89] Greek R. Shanks N. (2011). Complex systems, evolution, and animal models. *Stud. Hist. Philos. Biol. Biomed. Sci.* 42 542–544. 22035727 10.1016/j.shpsc.2011.07.001

[B90] Grzegorski T. Losy J. (2019). Multiple sclerosis - the remarkable story of a baffling disease. *Rev. Neurosci.* 30 511–526. 10.1515/revneuro-2018-0074 30645198

[B91] Hair K. Macleod M. R. Sena E. S. Collaboration I. I. (2019). A randomised controlled trial of an intervention to improve compliance with the ARRIVE guidelines (IICARus). *Res. Integr. Peer Rev.* 4:12. 10.1186/s41073-019-0069-3 PMC6560728 31205756

[B92] Hamilton S. E. Griffith T. S. (2019). A wild microbiome improves mouse modeling of the human immune response. *Lab Anim.* 48 337–338. 10.1038/s41684-019-0421-8 31591550

[B93] Hankey G. J. (2017). Stroke. *Lancet* 389 641–654. 27637676 10.1016/S0140-6736(16)30962-X

[B94] Hankey G. J. (2020). Nerinetide before reperfusion in acute ischaemic stroke: deja vu or new insights? *Lancet* 395 843–844. 10.1016/s0140-6736(20)30316-0 32087817

[B95] Hansen F. B. Secher N. Jensen M. S. Ostergaard L. Tonnesen E. Granfeldt A. (2017). Cortical spreading depolarizations in the postresuscitation period in a cardiac arrest male rat model. *J. Neurosci. Res.* 95 2040–2050. 10.1002/jnr.24033 28198552

[B96] Harriott A. M. Strother L. C. Vila-Pueyo M. Holland P. R. (2019). Animal models of migraine and experimental techniques used to examine trigeminal sensory processing. *J. Headache Pain* 20:91. 10.1186/s10194-019-1043-7 PMC6734323 31464579

[B97] Harrison J. K. Mcarthur K. S. Quinn T. J. (2013). Assessment scales in stroke: clinimetric and clinical considerations. *Clin. Interv. Aging* 8 201–211. 10.2147/cia.s32405 23440256 PMC3578502

[B98] t Hart B. A. Laman J. D. Kap Y. S. (2018). Merits and complexities of modeling multiple sclerosis in non-human primates: implications for drug discovery. *Expert Opin. Drug Discov.* 13 387–397. 10.1080/17460441.2018.1443075 29465302

[B99] Hausenloy D. J. Kharbanda R. K. Møller U. K. Ramlall M. Aarøe J. Butler R. (2019). Effect of remote ischaemic conditioning on clinical outcomes in patients with acute myocardial infarction (CONDI-2/ERIC-PPCI): a single-blind randomised controlled trial. *Lancet* 394 1415–1424. 31500849 10.1016/S0140-6736(19)32039-2 PMC6891239

[B100] Hay D. L. Garelja M. L. Poyner D. R. Walker C. S. (2018). Update on the pharmacology of calcitonin/CGRP family of peptides: IUPHAR review 25. *Br. J. Pharmacol.* 175 3–17. 10.1111/bph.14075 29059473 PMC5740251

[B101] Herculano-Houzel S. (2009). The human brain in numbers: a linearly scaled-up primate brain. *Front. Hum. Neurosci.* 3:31. 10.3389/neuro.09.031.2009 19915731 PMC2776484

[B102] Heusch G. (2013). Cardioprotection: chances and challenges of its translation to the clinic. *Lancet* 381 166–175. 10.1016/s0140-6736(12)60916-7 23095318

[B103] Heusch G. (2017). Critical issues for the translation of cardioprotection. *Circ. Res.* 120 1477–1486. 10.1161/circresaha.117.310820 28450365

[B104] Heusch G. (2019). Coronary microvascular obstruction: the new frontier in cardioprotection. *Basic Res. Cardiol.* 114:45. 10.1007/s00395-019-0756-8 31617010

[B105] Heusch G. Gersh B. J. (2017). The pathophysiology of acute myocardial infarction and strategies of protection beyond reperfusion: a continual challenge. *Eur. Heart J.* 38 774–784. 27354052 10.1093/eurheartj/ehw224

[B106] Hill M. D. Goyal M. Menon B. K. Nogueira R. G. Mctaggart R. A. Demchuk A. M. (2020). Efficacy and safety of nerinetide for the treatment of acute ischaemic stroke (ESCAPE-NA1): a multicentre, double-blind, randomised controlled trial. *Lancet* 395 878–887. 32087818 10.1016/S0140-6736(20)30258-0

[B107] Hochstrasser T. Jiangshan Z. Ruhling S. Schmitz C. Kipp M. (2018). Do pre-clinical multiple sclerosis models allow us to measure neurodegeneration and clinical progression? *Expert Rev. Neurother.* [Epub ahead of print]. 10.1080/14737175.2018.1459190 29589965

[B108] Hodge R. D. Bakken T. E. Miller J. A. Smith K. A. Barkan E. R. Graybuck L. T. (2019). Conserved cell types with divergent features in human versus mouse cortex. *Nature* 573 61–68. 31435019 10.1038/s41586-019-1506-7 PMC6919571

[B109] Hoffmann A. Stoffel M. H. Nitzsche B. Lobsien D. Seeger J. Schneider H. (2014). The ovine cerebral venous system: comparative anatomy, visualization, and implications for translational research. *PLoS One* 9:e92990. 10.1371/journal.pone.0092990 24736654 PMC3988027

[B110] Holman C. Piper S. K. Grittner U. Diamantaras A. A. Kimmelman J. Siegerink B. (2016). Where have all the rodents gone? The effects of attrition in experimental research on cancer and stroke. *PLoS Biol.* 14:e1002331. 10.1371/journal.pbio.1002331 26726833 PMC4699644

[B111] Honegger P. (2001). Overview of cell and tissue culture techniques. *Curr. Protoc. Pharmacol.* Chapter 12:Unit 12.11. 10.1002/0471141755.ph1201s04 21965066

[B112] Hotter B. Ulm L. Hoffmann S. Katan M. Montaner J. Bustamante A. (2017). Selection bias in clinical stroke trials depending on ability to consent. *BMC Neurol.* 17:206. 10.1186/s12883-017-0989-9 29202730 PMC5716230

[B113] Howells D. W. Porritt M. J. Rewell S. S. O’collins V. Sena E. S. Van Der Worp H. B. (2010). Different strokes for different folks: the rich diversity of animal models of focal cerebral ischemia. *J. Cereb. Blood Flow Metab.* 30 1412–1431. 10.1038/jcbfm.2010.66 20485296 PMC2949237

[B114] Howells D. W. Sena E. S. O’collins V. Macleod M. R. (2012). Improving the efficiency of the development of drugs for stroke. *Int. J. Stroke* 7 371–377. 10.1111/j.1747-4949.2012.00805.x 22712738

[B115] Huang W. Percie, Du Sert N. Vollert J. Rice A. S. C. (2020). “General principles of preclinical study design,” in *Good Research Practice in Non-Clinical Pharmacology and Biomedicine*, eds Bespalov A. Michel M. C. Steckler T. (Cham: Springer International Publishing), 55–69. 10.1007/164_2019_277 PMC7610693 31707471

[B116] Huber C. Huber M. Ding Y. (2019). Evidence and opportunities of hypothermia in acute ischemic stroke: clinical trials of systemic versus selective hypothermia. *Brain Circ.* 5 195–202. 10.4103/bc.bc_25_19 31950095 PMC6950508

[B117] Hypothermia after Cardiac Arrest Study Group (2002). Mild therapeutic hypothermia to improve the neurologic outcome after cardiac arrest. *N. Engl. J. Med.* 346 549–556. 10.1056/nejmoa012689 11856793

[B118] Iadecola C. Buckwalter M. S. Anrather J. (2020). Immune responses to stroke: mechanisms, modulation, and therapeutic potential. *J. Clin. Invest.* 130 2777–2788. 10.1172/jci135530 32391806 PMC7260029

[B119] Ibanez B. James S. Agewall S. Antunes M. Bucciarelli-Ducci C. Bueno H. (2018). 2017 ESC Guidelines for the management of acute myocardial infarction in patients presenting with ST-segment elevation: the Task Force for the management of acute myocardial infarction in patients presenting with ST-segment elevation of the European Society of Cardiology (ESC). *Eur. Heart J.* 39 119–177. 28886621 10.1093/eurheartj/ehx393

[B120] Ioannidis J. P. (2005). Why most published research findings are false. *PLoS Med.* 2:e124. 10.1371/journal.pmed.0020124 16060722 PMC1182327

[B121] Ishii A. Dutta R. Wark G. M. Hwang S. I. Han D. K. Trapp B. D. (2009). Human myelin proteome and comparative analysis with mouse myelin. *Proc. Natl. Acad. Sci. U.S.A.* 106 14605–14610. 10.1073/pnas.0905936106 19706548 PMC2732873

[B122] Ito R. Takahashi T. Ito M. (2018). Humanized mouse models: application to human diseases. *J. Cell. Physiol.* 233 3723–3728. 10.1002/jcp.26045 28598567

[B123] Jackson S. J. Andrews N. Ball D. Bellantuono I. Gray J. Hachoumi L. (2017). Does age matter? The impact of rodent age on study outcomes. *Lab. Anim.* 51 160–169. 10.1177/0023677216653984 27307423 PMC5367550

[B124] Jacobs S. E. Berg M. Hunt R. Tarnow-Mordi W. O. Inder T. E. Davis P. G. (2013). Cooling for newborns with hypoxic ischaemic encephalopathy. *Cochrane Database Syst. Rev.* 1:CD003311. 10.1002/14651858.CD003311 14583966

[B125] Jacobs S. E. Morley C. J. Inder T. E. Stewart M. J. Smith K. R. Mcnamara P. J. (2011). Whole-body hypothermia for term and near-term newborns with hypoxic-ischemic encephalopathy: a randomized controlled trial. *Arch. Pediatr. Adolesc. Med.* 165 692–700. 10.1001/archpediatrics.2011.43 21464374

[B126] Jones S. P. Tang X. L. Guo Y. Steenbergen C. Lefer D. J. Kukreja R. C. (2015). The NHLBI-sponsored consortium for preclinicAl assESsment of cARdioprotective therapies (CAESAR): a new paradigm for rigorous, accurate, and reproducible evaluation of putative infarct-sparing interventions in mice, rabbits, and pigs. *Circ. Res.* 116 572–586. 10.1161/circresaha.116.305462 25499773 PMC4329104

[B127] Kaiser E. E. West F. D. (2020). Large animal ischemic stroke models: replicating human stroke pathophysiology. *Neural Regen. Res.* 15 1377–1387. 10.4103/1673-5374.274324 31997796 PMC7059570

[B128] Kalmbach B. E. Buchin A. Long B. Close J. Nandi A. Miller J. A. (2018). h-Channels contribute to divergent intrinsic membrane properties of supragranular pyramidal neurons in human versus mouse cerebral cortex. *Neuron* 100 1194.e5–1208.e5. 30392798 10.1016/j.neuron.2018.10.012 PMC6447369

[B129] Katchanov J. Waeber C. Gertz K. Gietz A. Winter B. Bruck W. (2003). Selective neuronal vulnerability following mild focal brain ischemia in the mouse. *Brain Pathol.* 13 452–464. 10.1111/j.1750-3639.2003.tb00476.x 14655751 PMC8095859

[B130] Katz Sand I. (2015). Classification, diagnosis, and differential diagnosis of multiple sclerosis. *Curr. Opin. Neurol.* 28 193–205. 10.1097/wco.0000000000000206 25887774

[B131] Kaur J. Zhao Z. Klein G. M. Lo E. H. Buchan A. M. (2004). The neurotoxicity of tissue plasminogen activator? *J. Cereb. Blood Flow Metab.* 24 945–963. 15356416 10.1097/01.WCB.0000137868.50767.E8

[B132] Kawamura N. Schmelzer J. D. Wang Y. Schmeichel A. M. Low P. A. (2005). The therapeutic window of hypothermic neuroprotection in experimental ischemic neuropathy: protection in ischemic phase and potential deterioration in later reperfusion phase. *Exp. Neurol.* 195 305–312. 10.1016/j.expneurol.2005.05.005 15950971

[B133] Kernan W. N. Ovbiagele B. Black H. R. Bravata D. M. Chimowitz M. I. Ezekowitz M. D. (2014). Guidelines for the prevention of stroke in patients with stroke and transient ischemic attack: a guideline for healthcare professionals from the American Heart Association/American Stroke Association. *Stroke* 45 2160–2236. 24788967 10.1161/STR.0000000000000024

[B134] Kilkenny C. Browne W. J. Cuthill I. C. Emerson M. Altman D. G. (2010). Improving bioscience research reporting: the ARRIVE guidelines for reporting animal research. *PLoS Biol.* 8:e1000412. 10.1371/journal.pbio.1000412 20613859 PMC2893951

[B135] Kimmelman J. Mogil J. S. Dirnagl U. (2014). Distinguishing between exploratory and confirmatory preclinical research will improve translation. *PLoS Biol.* 12:e1001863. 10.1371/journal.pbio.1001863 24844265 PMC4028181

[B136] Kipp M. Nyamoya S. Hochstrasser T. Amor S. (2017). Multiple sclerosis animal models: a clinical and histopathological perspective. *Brain Pathol.* 27 123–137. 10.1111/bpa.12454 27792289 PMC8029141

[B137] Kleinbongard P. Bøtker H. E. Ovize M. Hausenloy D. J. Heusch G. (2019). Co-morbidities and co-medications as confounders of cardioprotection-Does it matter in the clinical setting? *Br. J. Pharmacol.* 177 5252–5269. 10.1111/bph.14839 31430831 PMC7680006

[B138] Kola I. Landis J. (2004). Can the pharmaceutical industry reduce attrition rates? *Nat. Rev. Drug Discov.* 3 711–715. 10.1038/nrd1470 15286737

[B139] Krieger D. W. Yenari M. A. (2004). Therapeutic hypothermia for acute ischemic stroke: what do laboratory studies teach us? *Stroke* 35 1482–1489. 10.1161/01.str.0000126118.44249.5c 15073396

[B140] Kringe L. Sena E. S. Motschall E. Bahor Z. Wang Q. Herrmann A. M. (2020). Quality and validity of large animal experiments in stroke: a systematic review. *J. Cereb. Blood Flow Metab.* 40 2152–2164. 10.1177/0271678x20931062 32576074 PMC7585919

[B141] Kuczynski A. M. Marzoughi S. Al Sultan A. S. Colbourne F. Menon B. K. Van Es A. (2020). Therapeutic hypothermia in acute ischemic stroke-a systematic review and meta-analysis. *Curr. Neurol. Neurosci. Rep.* 20:13. 10.1007/s11910-020-01029-3 32372297

[B142] Kumar A. Gupta V. J. B. R. B. (2016). A review on animal models of stroke: an update. *Brain Res. Bull.* 122 35–44. 10.1016/j.brainresbull.2016.02.016 26902651

[B143] Kvistad C. E. Thomassen L. Waje-Andreassen U. Naess H. (2012). Low body temperature associated with severe ischemic stroke within 6 hours of onset: the Bergen NORSTROKE Study. *Vasc. Health Risk Manag.* 8 333–338. 10.2147/vhrm.s31614 22701327 PMC3373317

[B144] La Rosa C. Bonfanti L. (2018). Brain plasticity in mammals: an example for the role of comparative medicine in the neurosciences. *Front. Vet. Sci.* 5:274. 10.3389/fvets.2018.00274 30443551 PMC6221904

[B145] Labat-gest V. Tomasi S. (2013). Photothrombotic ischemia: a minimally invasive and reproducible photochemical cortical lesion model for mouse stroke studies. *J. Vis. Exp.* 50370. 10.3791/50370 23770844 PMC3727176

[B146] Lancaster M. A. Renner M. Martin C. A. Wenzel D. Bicknell L. S. Hurles M. E. (2013). Cerebral organoids model human brain development and microcephaly. *Nature* 501 373–379. 23995685 10.1038/nature12517 PMC3817409

[B147] Lapchak P. A. (2010). Translational stroke research using a rabbit embolic stroke model: a correlative analysis hypothesis for novel therapy development. *Transl. Stroke Res.* 1 96–107. 10.1007/s12975-010-0018-4 20539748 PMC2881325

[B148] Lapchak P. A. Zhang J. H. Noble-Haeusslein L. J. (2013). RIGOR guidelines: escalating STAIR and STEPS for effective translational research. *Transl. Stroke Res.* 4 279–285. 10.1007/s12975-012-0209-2 23658596 PMC3644408

[B149] Lassmann H. Bradl M. (2017). Multiple sclerosis: experimental models and reality. *Acta Neuropathol.* 133 223–244. 10.1007/s00401-016-1631-4 27766432 PMC5250666

[B150] le Feber J. Tzafi Pavlidou S. Erkamp N. Van Putten M. J. Hofmeijer J. (2016). Progression of neuronal damage in an in vitro model of the ischemic penumbra. *PLoS One* 11:e0147231. 10.1371/journal.pone.0147231 26871437 PMC4752264

[B151] Leenaars C. H. C. Kouwenaar C. Stafleu F. R. Bleich A. Ritskes-Hoitinga M. De Vries R. B. M. (2019). Animal to human translation: a systematic scoping review of reported concordance rates. *J. Transl. Med.* 17:223. 10.1186/s12967-019-1976-2 PMC6631915 31307492

[B152] Lees K. R. Zivin J. A. Ashwood T. Davalos A. Davis S. M. Diener H. C. (2006). NXY-059 for acute ischemic stroke. *N. Engl. J. Med.* 354 588–600. 16467546 10.1056/NEJMoa052980

[B153] Li M. Izpisua Belmonte J. C. (2019). Organoids - preclinical models of human disease. *Reply N. Engl. J. Med.* 380 569–579. 10.1056/nejmra1806175 30726695

[B154] Li N. Kong X. Ye R. Yang Q. Han J. Xiong L. (2011). Age-related differences in experimental stroke: possible involvement of mitochondrial dysfunction and oxidative damage. *Rejuvenation Res.* 14 261–273. 10.1089/rej.2010.1115 21466386

[B155] Lindsey M. L. Bolli R. Canty J. M. Jr. Du X. J. Frangogiannis N. G. Frantz S. (2018). Guidelines for experimental models of myocardial ischemia and infarction. *Am. J. Physiol. Heart Circ. Physiol.* 314 H812–H838. 29351451 10.1152/ajpheart.00335.2017 PMC5966768

[B156] Liu S. (2009). Dealing with publication bias in translational stroke research. *J. Exp. Stroke Transl. Med.* 2 16–21. 10.6030/1939-067x-2.1.16 20431704 PMC2860750

[B157] Liu T. Zhao D. X. Cui H. Chen L. Bao Y. H. Wang Y. (2016). Therapeutic hypothermia attenuates tissue damage and cytokine expression after traumatic brain injury by inhibiting necroptosis in the rat. *Sci. Rep.* 6:24547. 10.1038/srep24547 PMC4832230 27080932

[B158] Llovera G. Hofmann K. Roth S. Salas-Perdomo A. Ferrer-Ferrer M. Perego C. (2015). Results of a preclinical randomized controlled multicenter trial (pRCT): Anti-CD49d treatment for acute brain ischemia. *Sci. Transl. Med.* 7:299ra121. 10.1126/scitranslmed.aaa9853 26246166

[B159] Lo E. H. (2014). 2013 thomas willis award lecture. *Stroke* 45 305–308. 10.1161/strokeaha.113.001269 24203848 PMC4067238

[B160] London A. J. Kimmelman J. (2015). Why clinical translation cannot succeed without failure. *eLife* 4:e12844. 10.7554/eLife.12844 PMC4657068 26599839

[B161] Lourbopoulos A. Karacostas D. Artemis N. Milonas I. Grigoriadis N. (2008). Effectiveness of a new modified intraluminal suture for temporary middle cerebral artery occlusion in rats of various weight. *J. Neurosci. Methods* 173 225–234. 10.1016/j.jneumeth.2008.06.018 18634824

[B162] Lourbopoulos A. Mamrak U. Roth S. Balbi M. Shrouder J. Liesz A. (2017). Inadequate food and water intake determine mortality following stroke in mice. *J. Cereb. Blood Flow Metab.* 37 2084–2097. 10.1177/0271678x16660986 27449604 PMC5464703

[B163] Lowenstein P. R. Castro M. G. (2009). Uncertainty in the translation of preclinical experiments to clinical trials. Why do most phase III clinical trials fail? *Curr. Gene Ther.* 9 368–374. 10.2174/156652309789753392 19860651 PMC2864134

[B164] Luders E. Steinmetz H. Jancke L. (2002). Brain size and grey matter volume in the healthy human brain. *Neuroreport* 13 2371–2374. 10.1097/00001756-200212030-00040 12488829

[B165] Ludman A. Yellon D. Hasleton J. Ariti C. Babu G. Boston-Griffiths E. (2011). Effect of erythropoietin as an adjunct to primary percutaneous coronary intervention: a randomised controlled clinical trial. *Heart* 97 1560–1565. 10.1136/hrt.2011.223867 21900585

[B166] Ma H. Sinha B. Pandya R. S. Lin N. Popp A. J. Li J. (2012). Therapeutic hypothermia as a neuroprotective strategy in neonatal hypoxic-ischemic brain injury and traumatic brain injury. *Curr. Mol. Med.* 12 1282–1296. 10.2174/156652412803833517 22834830 PMC4173077

[B167] Makin T. R. Orban de Xivry J. J. (2019). Ten common statistical mistakes to watch out for when writing or reviewing a manuscript. *eLife* 8:e48175. 10.7554/eLife.48175 PMC6785265 31596231

[B168] Mangion K. Carrick D. Clerfond G. Rush C. Mccomb C. Oldroyd K. (2019). Predictors of segmental myocardial functional recovery in patients after an acute ST-Elevation myocardial infarction. *Eur. J. Radiol.* 112 121–129. 30777200 10.1016/j.ejrad.2019.01.010 PMC6390173

[B169] Martic-Kehl M. I. Schibli R. Schubiger P. A. (2012). Can animal data predict human outcome? Problems and pitfalls of translational animal research. *Eur. J. Nucl. Med. Mol. Imaging* 39 1492–1496. 10.1007/s00259-012-2175-z 22790876 PMC3411287

[B170] May A. (2017). Understanding migraine as a cycling brain syndrome: reviewing the evidence from functional imaging. *Neurol. Sci.* 38 125–130. 10.1007/s10072-017-2866-0 28527054

[B171] Mergenthaler P. Meisel A. (2012). Do stroke models model stroke? *Dis. Model. Mech.* 5 718–725. 10.1242/dmm.010033 23115201 PMC3484854

[B172] Mering S. Jolkkonen J. (2015). Proper housing conditions in experimental stroke studies-special emphasis on environmental enrichment. *Front. Neurosci.* 9:106. 10.3389/fnins.2015.00106 25870536 PMC4378295

[B173] Mestas J. Hughes C. C. (2004). Of mice and not men: differences between mouse and human immunology. *J. Immunol.* 172 2731–2738. 10.4049/jimmunol.172.5.2731 14978070

[B174] Mestre H. Mori Y. Nedergaard M. (2020). The brain’s glymphatic system: current controversies. *Trends Neurosci.* 43 458–466. 10.1016/j.tins.2020.04.003 32423764 PMC7331945

[B175] Meuth S. G. Bittner S. Ulzheimer J. C. Kleinschnitz C. Kieseier B. C. Wiendl H. (2010). Therapeutic approaches to multiple sclerosis: an update on failed, interrupted, or inconclusive trials of neuroprotective and alternative treatment strategies. *BioDrugs* 24 317–330. 10.2165/11537190-000000000-00000 20795753

[B176] Miller S. Liu H. Warfvinge K. Shi L. Dovlatyan M. Xu C. (2016). Immunohistochemical localization of the calcitonin gene-related peptide binding site in the primate trigeminovascular system using functional antagonist antibodies. *Neuroscience* 328 165–183. 10.1016/j.neuroscience.2016.04.046 27155150

[B177] Modo M. M. Jolkkonen J. Zille M. Boltze J. (2018). Future of animal modeling for poststroke tissue repair. *Stroke* 49 1099–1106. 10.1161/strokeaha.117.018293 29669872 PMC6013070

[B178] Moskowitz M. A. Lo E. H. Iadecola C. (2010). The science of stroke: mechanisms in search of treatments. *Neuron* 67 181–198. 10.1016/j.neuron.2010.07.002 20670828 PMC2957363

[B179] Normand R. Du W. Briller M. Gaujoux R. Starosvetsky E. Ziv-Kenet A. (2018). Found in translation: a machine learning model for mouse-to-human inference. *Nat. Methods* 15 1067–1073. 10.1038/s41592-018-0214-9 30478323 PMC12396630

[B180] Norris D. Dirnagl U. Zigmond M. J. Thompson-Peer K. Chow T. T. (2018). Health tips for research groups. *Nature* 557 302–304. 10.1038/d41586-018-05146-5 29769687

[B181] Oberheim N. A. Takano T. Han X. He W. Lin J. H. Wang F. (2009). Uniquely hominid features of adult human astrocytes. *J. Neurosci.* 29 3276–3287. 10.1523/jneurosci.4707-08.2009 19279265 PMC2819812

[B182] O’Collins V. E. Macleod M. R. Donnan G. A. Horky L. L. Van Der Worp B. H. Howells D. W. (2006). 1,026 experimental treatments in acute stroke. *Ann. Neurol.* 59 467–477. 16453316 10.1002/ana.20741

[B183] Otani T. Marchetto M. C. Gage F. H. Simons B. D. Livesey F. J. (2016). 2D and 3D stem cell models of primate cortical development identify species-specific differences in progenitor behavior contributing to brain size. *Cell Stem Cell* 18 467–480. 10.1016/j.stem.2016.03.003 27049876 PMC4826446

[B184] Panchal A. R. Bartos J. A. Cabanas J. G. Donnino M. W. Drennan I. R. Hirsch K. G. (2020). Part 3: adult basic and advanced life support: 2020 American Heart Association guidelines for cardiopulmonary resuscitation and emergency cardiovascular care. *Circulation* 142 S366–S468. 33081529 10.1161/CIR.0000000000000916

[B185] Pantos C. Malliopoulou V. Varonos D. D. Cokkinos D. V. (2004). Thyroid hormone and phenotypes of cardioprotection. *Basic Res. Cardiol.* 99 101–120. 10.1007/s00395-003-0449-0 14963669

[B186] Pascual J. (2015). CGRP antibodies: the Holy Grail for migraine prevention? *Lancet Neurol.* 14 1066–1067. 10.1016/s1474-4422(15)00244-6 26432183

[B187] Patel S. D. Pierce L. Ciardiello A. Hutton A. Paskewitz S. Aronowitz E. (2015). Therapeutic hypothermia and hypoxia–ischemia in the term-equivalent neonatal rat: characterization of a translational preclinical model. *Pediatr. Res.* 78 264–271. 10.1038/pr.2015.100 25996893 PMC4543535

[B188] Patino C. M. Ferreira J. C. (2018). Internal and external validity: can you apply research study results to your patients? *J. Brasil. Pneumol.* 44 183–183. 10.1590/s1806-37562018000000164 30043882 PMC6188693

[B189] Pedersen F. Butrymovich V. Kelbaek H. Wachtell K. Helqvist S. Kastrup J. (2014). Short- and long-term cause of death in patients treated with primary PCI for STEMI. *J. Am. Coll. Cardiol.* 64 2101–2108. 10.1016/j.jacc.2014.08.037 25457398

[B190] Percie du Sert N. Alfieri A. Allan S. M. Carswell H. V. Deuchar G. A. (2017). The IMPROVE guidelines (Ischaemia models: procedural refinements of in vivo experiments). *J. Cereb. Blood Flow Metab.* 37 3488–3517. 10.1177/0271678x17709185 28797196 PMC5669349

[B191] Popa-Wagner A. Badan I. Walker L. Groppa S. Patrana N. Kessler C. (2007). Accelerated infarct development, cytogenesis and apoptosis following transient cerebral ischemia in aged rats. *Acta Neuropathol.* 113 277–293. 10.1007/s00401-006-0164-7 17131130

[B192] Powers W. J. Rabinstein A. A. Ackerson T. Adeoye O. M. Bambakidis N. C. Becker K. (2019). Guidelines for the early management of patients with acute ischemic stroke: 2019 update to the 2018 guidelines for the early management of acute ischemic stroke: a guideline for healthcare professionals from the American Heart Association/American Stroke Association. *Stroke* 50:00e344-18.

[B193] Pugsley M. K. (2002). The diverse molecular mechanisms responsible for the actions of opioids on the cardiovascular system. *Pharmacol. Ther.* 93 51–75. 10.1016/s0163-7258(02)00165-1 11916541

[B194] Purkayastha J. Lewis L. E. Bhat R. Y. Anusha K. M. (2016). Feasibility and safety of therapeutic hypothermia and short term outcome in neonates with hypoxic ischemic encephalopathy. *Indian J. Pediatr.* 83 175–177. 10.1007/s12098-015-1829-9 26141549

[B195] Rao R. Trivedi S. Vesoulis Z. Liao S. M. Smyser C. D. Mathur A. M. (2017). Safety and short-term outcomes of therapeutic hypothermia in preterm neonates 34-35 weeks gestational age with hypoxic-ischemic encephalopathy. *J. Pediatr.* 183 37–42. 10.1016/j.jpeds.2016.11.019 27979578 PMC5367984

[B196] Raya T. E. Gaballa M. Anderson P. Goldman S. (1997). Left ventricular function and remodeling after myocardial infarction in aging rats. *Am. J. Physiol.* 273 H2652–H2658. 9435600 10.1152/ajpheart.1997.273.6.H2652

[B197] Reinstadler S. Stiermaier T. Reindl M. Feistritzer H. Fuernau G. Eitel C. (2018). Intramyocardial haemorrhage and prognosis after ST-elevation myocardial infarction. *Eur. Heart J. Cardiovasc. Imaging* 20 138–146. 10.1093/ehjci/jey101 30165518

[B198] Richter S. H. Garner J. P. Wurbel H. (2009). Environmental standardization: cure or cause of poor reproducibility in animal experiments? *Nat. Methods* 6 257–261. 10.1038/nmeth.1312 19333241

[B199] Rink C. Christoforidis G. Abduljalil A. Kontzialis M. Bergdall V. Roy S. (2008). Minimally invasive neuroradiologic model of preclinical transient middle cerebral artery occlusion in canines. *Proc. Natl. Acad. Sci. U.S.A.* 105 14100–14105. 10.1073/pnas.0806678105 18779582 PMC2544585

[B200] Ritzel R. M. Lai Y. J. Crapser J. D. Patel A. R. Schrecengost A. Grenier J. M. (2018). Aging alters the immunological response to ischemic stroke. *Acta Neuropathol.* 136 89–110. 10.1007/s00401-018-1859-2 29752550 PMC6015099

[B201] Rolfes L. Pawlitzki M. Pfeuffer S. Huntemann N. Wiendl H. Ruck T. (2020). Failed, interrupted, or inconclusive trials on immunomodulatory treatment strategies in multiple sclerosis: update 2015-2020. *BioDrugs* 34 587–610. 10.1007/s40259-020-00435-w 32785877 PMC7519896

[B202] Rongvaux A. Willinger T. Martinek J. Strowig T. Gearty S. V. Teichmann L. L. (2014). Development and function of human innate immune cells in a humanized mouse model. *Nat. Biotechnol.* 32 364–372. 10.1038/nbt.2858 24633240 PMC4017589

[B203] Rosenzweig S. Carmichael S. T. (2013). Age-dependent exacerbation of white matter stroke outcomes: a role for oxidative damage and inflammatory mediators. *Stroke* 44 2579–2586. 10.1161/strokeaha.113.001796 23868277 PMC3791618

[B204] Rossello X. Yellon D. M. (2016). Cardioprotection: the disconnect between bench and bedside. *Circulation* 134 574–575. 10.1161/circulationaha.116.022829 27550967

[B205] Rosshart S. P. Herz J. Vassallo B. G. Hunter A. Wall M. K. Badger J. H. (2019). Laboratory mice born to wild mice have natural microbiota and model human immune responses. *Science* 365:eaaw4361. 10.1126/science.aaw4361 31371577 PMC7377314

[B206] Rosshart S. P. Vassallo B. G. Angeletti D. Hutchinson D. S. Morgan A. P. Takeda K. (2017). Wild mouse gut microbiota promotes host fitness and improves disease resistance. *Cell* 171 1015.e3–1028.e3. 29056339 10.1016/j.cell.2017.09.016 PMC6887100

[B207] Roubille F. Lairez O. Mewton N. Rioufol G. Ranc S. Sanchez I. (2012). Cardioprotection by clopidogrel in acute ST-elevated myocardial infarction patients: a retrospective analysis. *Basic Res. Cardiol.* 107:275. 10.1007/s00395-012-0275-3 22718009

[B208] Rubio-Beltran E. Correnti E. Deen M. Kamm K. Kelderman T. Papetti L. (2018). PACAP38 and PAC1 receptor blockade: a new target for headache? *J. Headache Pain* 19:64. 10.1186/s10194-018-0893-8 PMC6081277 30088106

[B209] Russell F. A. King R. Smillie S. J. Kodji X. Brain S. D. (2014). Calcitonin gene-related peptide: physiology and pathophysiology. *Physiol. Rev.* 94 1099–1142. 10.1152/physrev.00034.2013 25287861 PMC4187032

[B210] Saver J. L. (2006). Time is brain–quantified. *Stroke* 37 263–266. 10.1161/01.str.0000196957.55928.ab 16339467

[B211] Saver J. L. Starkman S. Eckstein M. Stratton S. J. Pratt F. D. Hamilton S. (2015). Prehospital use of magnesium sulfate as neuroprotection in acute stroke. *N. Engl. J. Med.* 372 528–536. 10.1056/nejmoa1408827 25651247 PMC4920545

[B212] Savitz S. I. Chopp M. Deans R. Carmichael T. Phinney D. Wechsler L. (2011). Stem cell therapy as an emerging paradigm for stroke (STEPS) II. *Stroke* 42 825–829. 10.1161/strokeaha.110.601914 21273569

[B213] Schmidt J. Gold R. Schonrock L. Zettl U. K. Hartung H. P. Toyka K. V. (2000). T-cell apoptosis in situ in experimental autoimmune encephalomyelitis following methylprednisolone pulse therapy. *Brain* 123(Pt 7), 1431–1441. 10.1093/brain/123.7.1431 10869055

[B214] Schneider H. Kruger P. Algra A. Hofmeijer J. Van Der Worp H. B. Juttler E. (2017). No benefits of hypothermia in patients treated with hemicraniectomy for large ischemic stroke. *Int. J. Stroke* 12 732–740. 10.1177/1747493017694388 28350280

[B215] Schulte-Herbruggen O. Klehmet J. Quarcoo D. Meisel C. Meisel A. (2006). Mouse strains differ in their susceptibility to poststroke infections. *Neuroimmunomodulation* 13 13–18. 10.1159/000092109 16612133

[B216] Schuster N. M. Rapoport A. M. (2016). New strategies for the treatment and prevention of primary headache disorders. *Nat. Rev. Neurol.* 12 635–650. 10.1038/nrneurol.2016.143 27786243

[B217] Schuster N. M. Rapoport A. M. (2017). Calcitonin gene-related peptide-targeted therapies for migraine and cluster headache: a review. *Clin. Neuropharmacol.* 40 169–174. 10.1097/wnf.0000000000000227 28644160

[B218] Sekhon M. S. Ainslie P. N. Griesdale D. E. (2017). Clinical pathophysiology of hypoxic ischemic brain injury after cardiac arrest: a “two-hit” model. *Crit. Care* 21:90. 10.1186/s13054-017-1670-9 PMC5390465 28403909

[B219] Sena E. S. Van Der Worp H. B. Bath P. M. Howells D. W. Macleod M. R. (2010a). Publication bias in reports of animal stroke studies leads to major overstatement of efficacy. *PLoS Biol.* 8:e1000344. 10.1371/journal.pbio.1000344 20361022 PMC2846857

[B220] Sena E. S. Van Der Worp H. B. Bath P. M. W. Howells D. W. Macleod M. R. (2010b). Publication bias in reports of animal stroke studies leads to major overstatement of efficacy. *PLoS Biol.* 8:e1000344. 10.1371/journal.pbio.1000344 PMC2846857 20361022

[B221] Shah T. A. Nejad J. E. Pallera H. K. Lattanzio F. A. Farhat R. Kumar P. S. (2017). Therapeutic hypothermia modulates complement factor C3a and C5a levels in a rat model of hypoxic ischemic encephalopathy. *Pediatr. Res.* 81 654–662. 10.1038/pr.2016.271 28002390

[B222] Shanks N. Greek R. Greek J. (2009). Are animal models predictive for humans? *Philos Ethics Humanit Med.* 4:2. 10.1186/1747-5341-4-2 PMC2642860 19146696

[B223] Shuaib A. Lees K. R. Lyden P. Grotta J. Davalos A. Davis S. M. (2007). NXY-059 for the treatment of acute ischemic stroke. *N. Engl. J. Med.* 357 562–571. 17687131 10.1056/NEJMoa070240

[B224] Silveira R. C. Procianoy R. S. (2015a). Hipotermia terapêutica para recém-nascidos com encefalopatia hipóxico isquêmica. *J. Pediatr.* 91 S78–S83.

[B225] Silveira R. C. Procianoy R. S. (2015b). Hypothermia therapy for newborns with hypoxic ischemic encephalopathy. *J. Pediatr.* 91 S78–S83. 10.1016/j.jped.2015.07.004 26354871

[B226] Sims N. R. (1992). Energy metabolism and selective neuronal vulnerability following global cerebral ischemia. *Neurochem. Res.* 17 923–931. 10.1007/bf00993269 1407279

[B227] Sloth A. D. Schmidt M. R. Munk K. Schmidt M. Pedersen L. Sorensen H. T. (2015). Impact of cardiovascular risk factors and medication use on the efficacy of remote ischaemic conditioning: post hoc subgroup analysis of a randomised controlled trial. *BMJ Open* 5:e006923. 10.1136/bmjopen-2014-006923 25838505 PMC4390720

[B228] Sommer C. J. (2017). Ischemic stroke: experimental models and reality. *Acta Neuroparthol.* 133 245–261. 10.1007/s00401-017-1667-0 28064357 PMC5250659

[B229] Sorby-Adams A. J. Vink R. Turner R. J. (2018). Large animal models of stroke and traumatic brain injury as translational tools. *Am. J. Physiol. Regul. Integ. Comp. Physiol.* 315 R165–R190. 10.1152/ajpregu.00163.2017 29537289

[B230] Steffen J. Krohn M. Paarmann K. Schwitlick C. Bruning T. Marreiros R. (2016). Revisiting rodent models: octodon degus as Alzheimer’s disease model? *Acta Neuropathol. Commun.* 4:91. 10.1186/s40478-016-0363-y PMC5002178 27566602

[B231] Steimberg N. Bertero A. Chiono V. Dell’era P. Di Angelantonio S. Hartung T. (2020). iPS, organoids and 3D models as advanced tools for in vitro toxicology. *ALTEX* 37 136–140. 10.14573/altex.1911071 31960938

[B232] Steiner T. J. Stovner L. J. Birbeck G. L. (2013). Migraine: the seventh disabler. *Cephalalgia* 33 289–290. 10.1177/0333102412473843 23307815

[B233] Steinman L. Zamvil S. S. (2005). Virtues and pitfalls of EAE for the development of therapies for multiple sclerosis. *Trends Immunol.* 26 565–571. 10.1016/j.it.2005.08.014 16153891

[B234] Stone G. W. Selker H. P. Thiele H. Patel M. R. Udelson J. E. Ohman E. M. (2016). Relationship between infarct size and outcomes following primary PCI: patient-level analysis from 10 randomized trials. *J. Am. Coll. Cardiol.* 67 1674–1683. 10.1016/j.jacc.2016.01.069 27056772

[B235] Stortz J. A. Raymond S. L. Mira J. C. Moldawer L. L. Mohr A. M. Efron P. A. (2017). Murine models of sepsis and trauma: can we bridge the gap? *ILAR J.* 58 90–105. 10.1093/ilar/ilx007 28444204 PMC5886315

[B236] Stroke Unit Trialists’ Collaboration (2013). Organised inpatient (stroke unit) care for stroke. *Cochrane Database Syst. Rev.* 2013:CD000197. 10.1002/14651858.CD000197.pub3 PMC6474318 24026639

[B237] Sunwoldt J. Bosche B. Meisel A. Mergenthaler P. (2017). Neuronal culture microenvironments determine preferences in bioenergetic pathway use. *Front. Mol. Neurosci.* 10:305. 10.3389/fnmol.2017.00305 29085280 PMC5649214

[B238] Tagin M. A. Woolcott C. G. Vincer M. J. Whyte R. K. Stinson D. A. (2012). Hypothermia for neonatal hypoxic ischemic encephalopathy: an updated systematic review and meta-analysis. *Arch. Pediatr. Adolesc. Med.* 166 558–566. 22312166 10.1001/archpediatrics.2011.1772

[B239] Thomas A. Detilleux J. Flecknell P. Sandersen C. (2017). Impact of stroke therapy academic industry roundtable (STAIR) guidelines on peri-anesthesia care for rat models of stroke: a meta-analysis comparing the years 2005 and 2015. *PLoS One* 12:e0170243. 10.1371/journal.pone.0170243 28122007 PMC5266292

[B240] Thoresen M. Bågenholm R. Løberg E. Apricena F. Kjellmer I. (1996a). Posthypoxic cooling of neonatal rats provides protection against brain injury. *Arch. Dis. Child. Fetal Neonatal Ed.* 74 F3–F9. 8653432 10.1136/fn.74.1.f3 PMC2528334

[B241] Thoresen M. Bågenholm R. Løberg E. M. Apricena F. Kjellmer I. (1996b). Posthypoxic cooling of neonatal rats provides protection against brain injury. *Archives of Disease in Childhood - Fetal and Neonatal Edition*, 74 F3. 10.1136/fn.74.1.f3 PMC2528334 8653432

[B242] Titus H. E. Chen Y. Podojil J. R. Robinson A. P. Balabanov R. Popko B. (2020). Pre-clinical and clinical implications of “Inside-Out” vs. “Outside-In” paradigms in multiple sclerosis etiopathogenesis. *Front. Cell. Neurosci.* 14:599717. 10.3389/fncel.2020.599717 33192332 PMC7654287

[B243] Traystman R. J. (2003). Animal models of focal and global cerebral ischemia. *ILAR J.* 44 85–95. 10.1093/ilar.44.2.85 12652003

[B244] Truettner J. S. Alonso O. F. Dietrich W. D. (2005). Influence of therapeutic hypothermia on matrix metalloproteinase activity after traumatic brain injury in rats. *J. Cereb. Blood Flow Metab.* 25 1505–1516. 10.1038/sj.jcbfm.9600150 15959464

[B245] Tsivgoulis G. Alexandrov A. V. (2014). Does “time is brain” also mean “time is clot”? Time dependency of tissue-type plasminogen activator-induced recanalization in acute ischemic stroke. *Stroke* 45 2555–2556. 25104843 10.1161/STROKEAHA.114.006579

[B246] Uchino H. Ogihara Y. Fukui H. Chijiiwa M. Sekine S. Hara N. (2016). Brain injury following cardiac arrest: pathophysiology for neurocritical care. *J. Intensive Care* 4:31. 10.1186/s40560-016-0140-9 PMC4847238 27123307

[B247] van der Bijl P. Abou R. Goedemans L. Gersh B. Holmes D. J. Ajmone Marsan N. (2020). Left ventricular post-infarct remodeling: implications for systolic function improvement and outcomes in the modern Era. *JACC Heart Fail.* 8 131–140. 31838030 10.1016/j.jchf.2019.08.014

[B248] van Kranenburg M. Magro M. Thiele H. De Waha S. Eitel I. Cochet A. (2014). Prognostic value of microvascular obstruction and infarct size, as measured by CMR in STEMI patients. *J. Am. Coll. Cardiol. Cardiovasc. Imaging* 7 930–939. 10.1016/j.jcmg.2014.05.010 25212799

[B249] Velagaleti R. Pencina M. Murabito J. Wang T. Parikh N. D’agostino R. (2008). Long-term trends in the incidence of heart failure after myocardial infarction. *Circulation* 118 2057–2062. 18955667 10.1161/CIRCULATIONAHA.108.784215 PMC2729712

[B250] Vesterinen H. M. Sena E. S. Ffrench-Constant C. Williams A. Chandran S. Macleod M. R. (2010). Improving the translational hit of experimental treatments in multiple sclerosis. *Mult. Scler.* 16 1044–1055. 20685763 10.1177/1352458510379612

[B251] Vilela P. Rowley H. A. (2017). Brain ischemia: CT and MRI techniques in acute ischemic stroke. *Eur. J. Radiol.* 96 162–172. 29054448 10.1016/j.ejrad.2017.08.014

[B252] Vina J. Sanz-Ros J. (2018). Alzheimer’s disease: only prevention makes sense. *Eur. J. Clin. Invest.* 48:e13005. 10.1111/eci.13005 30028503

[B253] Vu Q. Xie K. Eckert M. Zhao W. Cramer S. C. (2014). Meta-analysis of preclinical studies of mesenchymal stromal cells for ischemic stroke. *Neurology* 82 1277–1286. 24610327 10.1212/WNL.0000000000000278 PMC4001201

[B254] Wang Y. Liu G. Hong D. Chen F. Ji X. Cao G. (2016). White matter injury in ischemic stroke. *Prog. Neurobiol.* 141 45–60. 27090751 10.1016/j.pneurobio.2016.04.005 PMC5677601

[B255] Watanabe H. Sakoh M. Andersen F. Rodell A. Sørensen J. C. Østergaard L. (2007). Statistical mapping of effects of middle cerebral artery occlusion (MCAO) on blood flow and oxygen consumption in porcine brain. *J. Neurosci. Methods* 160 109–115. 17129609 10.1016/j.jneumeth.2006.08.016

[B256] Wattiez A. S. Wang M. Russo A. F. (2019). CGRP in animal models of migraine. *Handb. Exp. Pharmacol.* 255 85–107. 30689086 10.1007/164_2018_187 PMC7008000

[B257] Welbourn C. Efstathiou N. (2018). How does the length of cardiopulmonary resuscitation affect brain damage in patients surviving cardiac arrest? A systematic review. *Scand. J. Trauma Resusc. Emerg. Med.* 26:77. 10.1186/s13049-018-0476-3 PMC6131783 30201018

[B258] Wells A. J. Vink R. Blumbergs P. C. Brophy B. P. Helps S. C. Knox S. J. (2012). A surgical model of permanent and transient middle cerebral artery stroke in the sheep. *PLoS One* 7:e42157. 10.1371/journal.pone.0042157 22848737 PMC3407087

[B259] Winek K. Dirnagl U. Meisel A. (2016). The gut microbiome as therapeutic target in central nervous system diseases: implications for stroke. *Neurotherapeutics* 13 762–774. 27714645 10.1007/s13311-016-0475-x PMC5081128

[B260] Wu L. Wu D. Yang T. Xu J. Chen J. Wang L. (2020). Hypothermic neuroprotection against acute ischemic stroke: The 2019 update. *J. Cereb. Blood Flow Metab.* 40 461–481. 31856639 10.1177/0271678X19894869 PMC7026854

[B261] Wyss-Coray T. (2016). Ageing, neurodegeneration and brain rejuvenation. *Nature* 539 180–186. 27830812 10.1038/nature20411 PMC5172605

[B262] Xinaris C. (2019). Organoids for replacement therapy: expectations, limitations and reality. *Curr. Opin. Organ Transplant.* 24 555–561. 31356234 10.1097/MOT.0000000000000680

[B263] Yamout B. Sahraian M. Bohlega S. Al-Jumah M. Goueider R. Dahdaleh M. (2020). Consensus recommendations for the diagnosis and treatment of multiple sclerosis: 2019 revisions to the MENACTRIMS guidelines. *Mult. Scler. Relat. Disord.* 37:101459. 10.1016/j.msard.2019.101459 31670208

[B264] Yao V. Kaletsky R. Keyes W. Mor D. E. Wong A. K. Sohrabi S. (2018). An integrative tissue-network approach to identify and test human disease genes. *Nat. Biotechnol.* 36 1091–1099. 10.1038/nbt.4246 PMC7021177 30346941

[B265] Yarborough M. Bredenoord A. D’abramo F. Joyce N. C. Kimmelman J. Ogbogu U. (2018). The bench is closer to the bedside than we think: uncovering the ethical ties between preclinical researchers in translational neuroscience and patients in clinical trials. *PLoS Biol.* 16:e2006343. 10.1371/journal.pbio.2006343 29874243 PMC6005633

[B266] Yarborough M. Dirnagl U. (2017). Preclinical research: Meet patients to sharpen up research. *Nature* 551 300. 10.1038/d41586-017-06024-2 29144480

[B267] Yetgin T. Uitterdijk A. Te Lintel, Hekkert M. Merkus D. Krabbendam-Peters I. (2015). Limitation of infarct size and no-reflow by intracoronary adenosine depends critically on dose and duration. *J. Am. Coll. Cardiol. Cardiovasc. Interv.* 8 1990–1999. 10.1016/j.jcin.2015.08.033 26738671

[B268] Yildiz E. P. Ekici B. Tatli B. (2017). Neonatal hypoxic ischemic encephalopathy: an update on disease pathogenesis and treatment. *Expert Rev. Neurother.* 17 449–459. 27830959 10.1080/14737175.2017.1259567

[B269] Zeiss C. J. Johnson L. K. (2017). Bridging the gap between reproducibility and translation: data resources and approaches. *ILAR J.* 58 1–3. 28586416 10.1093/ilar/ilx017

